# Non-invasive in vivo acoustoelectric neuromodulation and its contribution to ultrasound stimulation

**DOI:** 10.1038/s41467-026-73826-2

**Published:** 2026-06-17

**Authors:** Jean L. Rintoul, Christopher Butler, Robin O. Cleveland, Nir Grossman

**Affiliations:** 1 Department of Brain Sciences, Imperial College London, London, UK; 2 The George Institute for Global Health, School of Public Health, Imperial College London, London, UK; 3 Institute of Biomedical Engineering, University of Oxford, Oxford, UK

**Keywords:** Biomedical engineering, Electrophysiology

## Abstract

Non-invasive brain stimulation offers therapeutic potential without surgery, yet existing electrical approaches lack spatial precision due to the long wavelengths of electric fields. Here we demonstrate acoustoelectric neuromodulation, a nonlinear interaction between applied acoustic and electric fields that generates spatially localised, low-frequency electric fields at the ultrasound focus. Using in vitro and in vivo mouse electrophysiology, we show motor-evoked responses that depend on both the amplitude and frequency of the acoustoelectric field, with controls excluding purely acoustic or electrical origins. In vivo measurements show acoustoelectric potentials of ≈9 mV, corresponding to estimated focal electric fields of ~6 V/m at 500 kHz and 1 MPa acoustic pressure, with ~1.5 mm extrema spacing demonstrated in phantom experiments. Importantly, we identify an acoustoelectric contribution to conventional ultrasound stimulation, arising from interactions between ultrasound-induced electrical signals and propagating acoustic waves, establishing acoustoelectric neuromodulation as a distinct mechanism influencing ultrasound-based brain stimulation.

## Abstract (web summary)

Rintoul and colleagues show that ultrasound and electric fields can interact in the brain to generate low-frequency electric fields that drive neuromodulation, identifying an acoustoelectric contribution to ultrasound brain stimulation.

## Introduction

Non-invasive neuromodulation requires new approaches to achieve its translational potential. Electrical modalities - transcranial magnetic stimulation (TMS)^1^ and transcranial electrical stimulation (TES)^2–5^, span a continuum from established practice to rapidly advancing translational innovations, yet all are limited in spatial focality by the long wavelengths of electric fields and their uneven distribution in the brain’s heterogeneous dielectric structure.

In contrast, transcranial focused ultrasound stimulation (tFUS)^6^ offers superior spatial precision among non-invasive modalities, yet the mechanism by which acoustic fields modulate neuronal activity remains incompletely understood. Clarifying this mechanism is essential for improving specificity and safety. Proposed explanations include activation of mechanosensitive ion channels^7^, modulation of microtubules^8^, thermally mediated effects^9^, indirect auditory pathways^10^, cavitation^11^ and the acoustic radiation force^12^. By comparison, electric-field–driven modulation of neuronal excitability is mechanistically described within cable theory and Hodgkin–Huxley^13^ models. Ideally, a neuromodulation technique would be non-invasive, safe, elicit predictable responses at relevant amplitudes, and be selectively targetable to cortical and subcortical regions.

The acoustoelectric effect—the interaction of acoustic and electric fields in ionic media such as saline or brain tissue—may enable electrically mediated neuromodulation with the spatial precision of ultrasound. First described in 1946 by Fox, Herzfeld, and Rock^14^, the phenomenon arises from conductivity changes produced by compression and rarefaction of ions at the acoustic focus. When an electric field is present, these local conductivity fluctuations induce voltage gradients at the ultrasound focal zone. This principle underlies ultrasound current-source-density imaging^15,16^, enabling millimetre-scale spatial mapping of current distributions in cardiac tissue using the acoustoelectric effect. We have shown that the acoustoelectric effect is multiplicative (heterodyning)^17^, generating sum- and difference-frequency electric components confined to the acoustic focus and at physiologically relevant frequencies^18^.

**Fig. 1:**
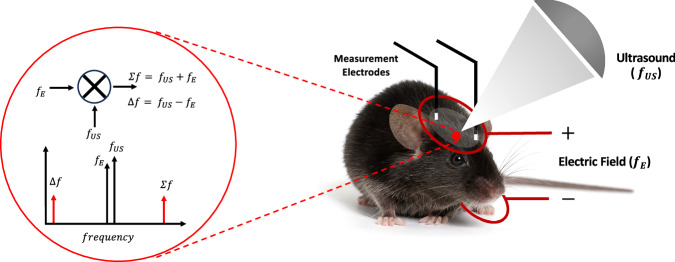
Acoustoelectric neuromodulation and its contribution to ultrasound neuromodulation. A focal acoustic field *P*(*f*_US_) and a non-focal electric field *E*(*f*_E_) mix via the acoustoelectric interaction, producing electrical signals at the difference frequency *f*_US_−*f*_E_ and at the sum frequency *f*_US_ + *f*_E_. If the difference frequency ∆*f* is in the range to induce electrophysiological responses, then neuromodulation is possible. Mouse photograph licensed from Shutterstock, image ID 160253894, ©Michiel de Wit/Shutterstock, used under Standard License.

If the difference frequency falls within electrophysiologically relevant frequencies and amplitudes, the resulting focused electric field could drive neuronal activation via a direct electric mechanism while preserving ultrasound’s focality (Fig. 1). This approach offers two independent control axes—acoustic and electric—for optimising amplitude and safety and permits ex vivo calibration by measuring the spatial profile and magnitude of the induced field.

In parallel, the voltage waveform that powers the ultrasound transducer can capacitively couple to surrounding ionic media (e.g., saline or brain tissue), generating secondary electrical artefacts at the ultrasonic frequency^19–21^. This coupling is enhanced when a water-filled coupling cone is positioned near the brain. We hypothesised that such artefactual fields can also participate in acoustoelectric mixing, producing a low-frequency component that contributes to tFUS-evoked responses.

Here, we test whether acoustoelectrically generated electric fields modulate neural activity in vitro (saline phantoms) and in vivo (mice). We find that evoked responses depend on both the amplitude and frequency of the acoustoelectric field and require the concurrent presence of acoustic and electric fields. Having established that acoustoelectric neuromodulation is genuine rather than artefactual, we further show that mixing between the transducer-driven artefactual field and the propagating acoustic wave is a significant contributor to ultrasound-induced brain stimulation. These findings identify the acoustoelectric effect as a key mechanism in ultrasound neuromodulation and support the development of safer, more precise, and mechanistically grounded non-invasive brain-stimulation therapies.

## Results

### Acoustoelectric amplitude characterisation

**Fig. 2:**
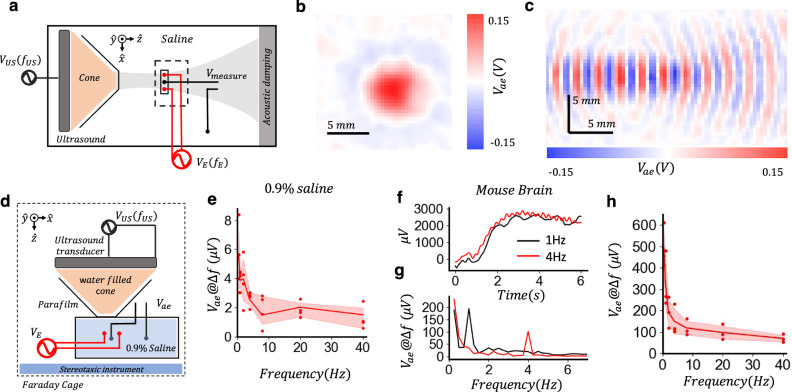
Amplitude characterisation of the acoustoelectric signal. **a** Free-field phantom measurements using a tank filled with 0.9% saline, a focused ultrasound transducer (500 kHz) generating a focal peak 1 MPa, two electrodes separated by 7 mm generating an electric field, and a measurement electrode. **b** $\hat{x}\hat{y}$ spatial map of acoustoelectric field (for ${V}_{E}$ = 12 V and ${f}_{E}$ = 8 kHz) band-filtered at ∆*f* = 492 kHz. **c** $\hat{x}\hat{z}$ spatial maps of acoustoelectric signal at ∆*f* as in (**b**). **d** In vitro setup using a petri dish with 0.9% saline to mimic the small volume of a mouse head, within the electrophysiology set up. **e** Measurements of ${V}_{{AE}}$ as a function of difference frequency (for ${V}_{E}$ = 10 V), data are presented as mean values ± s.d.; *n* = 4 measurements at each frequency. **f** In vivo mouse brain measurements using an electrode arrangement and settings as in e. Representative time series acoustoelectric amplitudes for ∆*f* = 1 Hz and ∆*f* = 4 Hz. **g** Amplitude spectrum of the acoustoelectric signal for ∆*f* = 1 Hz and ∆*f* = 4 Hz. **h** in vivo acoustoelectric amplitudes as a function of ∆*f*, Data are presented as mean values ± s.d.; *n* = 3 mice at each frequency. Source data are provided as a Source Data file.

First, we confirmed the focality of the acoustoelectrically generated electrical difference frequency in a tank filled with 0.9% saline (Fig. 2a), by using electrodes to generate an electric field and a 500 kHz focused transducer to create a focused ultrasound field at 1 MPa. A spatial map of the acoustoelectric field at $\Delta f$ was measured by translating the stimulating electrode pair and measurement electrode through the acoustic field with 0.5 mm steps. It can be seen that the acoustoelectric field in the lateral plane $\hat{x}\hat{y}$ (Fig. 2b) and axial plane $\hat{x}\hat{z}$ (Fig. 2c) is consistent with the focal region (Supplementary Fig. S1).

The acoustoelectric interaction has previously been reported with very small amplitudes^17,22^ that would be sub-threshold to neural responses^23^. However, it has primarily been explored at higher frequencies close to the ultrasound frequency^24^, leaving a knowledge gap at the low frequencies (0–100 Hz) known to stimulate neurons^25^. To understand acoustoelectric amplitudes at low frequencies, we measured the acoustoelectric voltage amplitudes over a range of low frequencies in a saline filled petri-dish within the intended in vivo electrophysiology apparatus (Fig. 2d) with the instrumentation detailed in **Methods**. We induced acoustoelectric difference frequencies within the range reported for motor stimulation^23,26^, by applying an electric field that was close in frequency to the propagating acoustic field, which remained the same. We found that acoustoelectric amplitudes increased as $\Delta f$ decreased (Fig. 2e).

Then, a recovery surgery was performed to insert two platinum-iridium electrodes connected to a small plug at the back of the mouse head so that the signal in the brain at the motor cortex could be measured in 3 wild type mice (see **Methods** for surgery details), using the same platinum-iridium electrodes and spacing as the saline petri-dish experiments. After surgery recovery, each mouse was anaesthetised with ketamine/xylazine and the same electrode and applied fields as the petri-dish were applied to the mouse brain. We show representative acoustoelectric time-series traces for Δf = 1 Hz and Δf = 4 Hz (Fig. 2f), where the electrical difference frequency is present alongside a DC offset. The corresponding spectral peaks (Fig. 2g) show the relative amplitude at 1 Hz was higher than at 4 Hz, when the applied electric and acoustic field amplitudes were the same. In vivo acoustoelectric amplitudes decreased with increasing $\Delta f$ (Fig. 2h, *n* = 3 mice), suggesting acoustoelectric neuromodulation would be most likely where the difference frequency between the applied fields was smallest.

### In vivo acoustoelectric DC neuromodulation

**Fig. 3:**
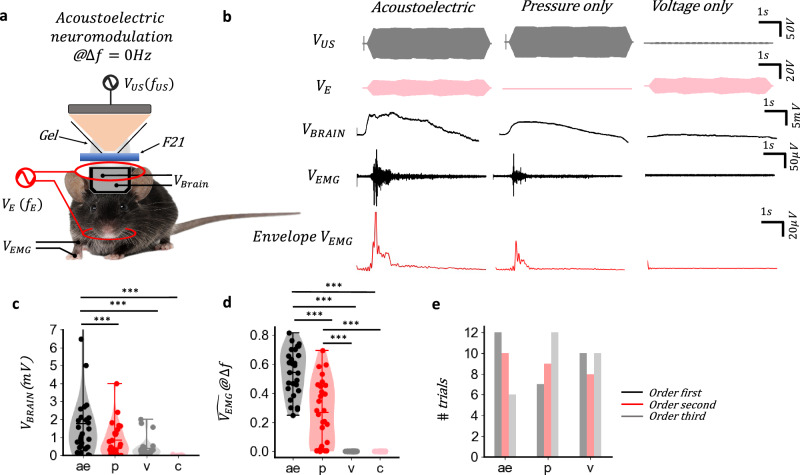
In vivo acoustoelectric amplitude neuromodulation @ Δ*f* = 0*Hz*. **a** In vivo arrangement to apply ultrasound while electrically shielding the RF artefact from the ultrasound with F21, with applied bipolar electrical stimulus via external ring electrodes. Acoustic and electric field are applied at 500 kHz. Mouse photograph licensed from Shutterstock, image ID 160253894, ©Michiel de Wit/Shutterstock, used under Standard License. **b** Illustrative example counterbalanced triplet (*P* = 2.5 MPa, ${V}_{E}$ = 20 V) showing ${V}_{{BRAIN}},{V}_{{EMG}}$ and the envelope of ${V}_{{EMG}}$. Acoustoelectric ${V}_{{BRAIN}}$ = 9.10 ${mV}$ peak onset, pressure only ${V}_{{BRAIN}}$ = 4.99 ${mV}$ and voltage only ${V}_{{BRAIN}}$ = 1.11 ${mV}$. ${V}_{{EMG}}$ software filtered between 100-1000 Hz, Envelope of ${V}_{{EMG}}$ low-pass filtered at 10 Hz. **c** Triplets applied in randomised order to account for anaesthesia depth changes. ${V}_{{BRAIN}}$ comparison between acoustoelectric, pressure only, voltage only and no stimulation control conditions. Data are presented as mean values ± s.d.; individual points represent triplet measurements with n = 28 triplets from 9 mice. Groups were compared using one way ANOVA followed by Tukey’s HSD post hoc test for post hoc pairwise comparisons: F(3,27) = 8.50, *p* = 4.8e-5, AE–P, *p* = 0.02; AE-V, *p* = 0.0003; P-V, *p* = 0.52; AE-C, *p* = 0.00; P-C, *p* = 0.21; V-C, *p* = 0.80. **d** Same dataset as in c, showing Hilbert EMG height normalised across each triplet, denoted by $\hat{{V}_{{EMG}}}$. Data are presented as mean values ± s.d.; with *n* = 28 triplets from 9 mice, statistics on the log ratios; ANOVA F(3,27) = 252.16, *p* = 0.0, Tukey HSD post hoc pairwise comparisons, AE–P, *p* = 0.002; AE-V, *p* = 0.0; AE-C, *p* = 0.0, P-V, *p* = 0.0, P-C, *p* < 0.0, V-C, *p* = 1.0. **e** Order analysis showing the number of acoustoelectric (AE), pressure (P) and voltage only trials and the order in the triplet. Chi-squared test Χ^2^ (4, *N* = 28) = 5.5, *p* = 0.23; Bonferroni corrected pairs; AE–P, *p* = 0.19; AE-V, *p* = 0.45; P-V, *p* = 0.54. Source data are provided as a Source Data file.

The same recovery surgery as described in Fig. 2 was performed in 9 wild-type mice, where each mouse was anaesthetised with ketamine/xylazine and the stimulation experiment conducted during the lightening anaesthesia window so that electromyography (EMG) responses could be measured in parallel to the evoked acoustoelectric field in the brain (see “**Methods**” for experiment details). Two platinum-iridium ring electrodes (diameter 2 cm) applied the bipolar electric field, and the ultrasound transducer was positioned via stereotaxic instrument over the head of the mouse (Fig. 3a) with two electrodes placed in the forepaw to measure neural evoked muscle responses (EMG), with electrically-attenuating acoustically-semi-transparent material (F21 characterised in Supplementary Fig. S2) placed between the transducer cone and gel creating an acoustic connection while reducing the electrical interference from the electrical transducer artefact^27^.

A series of three tests were performed to determine the effect of applying an acoustic or electric field alone and then both together, referred to as a triplet. Within each triplet, the voltage and pressure were the same, and each trial was 6 s long with a 0.25 s onset and offset ramp. The electric potential induced in the brain at $\Delta{\boldsymbol{f}}$ estimates the strength of the neuromodulatory electrical field, and the EMG potential in the paw estimates the strength of the evoked motor response. When both fields were applied together, and at the same frequency (500 kHz), an acoustoelectric difference frequency $\Delta{\boldsymbol{f}}$ was measurable in the brain at 0 Hz (DC). This low-frequency electric field typically had a steep gradient at the onset, shown by the example recordings in Fig. 3b and Supplementary Video 1. Simultaneous EMG recording shows the direct muscle response at the onset and how it differs between the three different stimuli, with the EMG envelope computed via Hilbert transform and then low-pass filtered at 10 Hz.

To overcome the changing sensitivity to the applied stimulus due to the anaesthesia depth changes, we applied each triplet in counterbalanced order (see Supplementary Fig. S3 on impact of anaesthesia on evoked EMG amplitudes) over 9 mice with 3 or 4 triplets per mouse yielding a total of 28 trials, where the only data selection criterion was that the anaesthesia should be light enough to evoke an EMG response in any trial of the triplet. An important additional question is whether the acoustic or electric fields induced a neuromodulation response alone. We found that the DC electric potential induced in the brain by the acoustoelectric stimulation was larger than that induced by the acoustic or electric stimulation alone, and larger than a no stimulus control (ANOVA F(3,27) = 8.50, *p* = 4.8e-5; Fig. 3c). Similarly, the normalised EMG amplitude^27^ induced in the paw by the acoustoelectric stimulation was larger than the those induced by pressure or voltage alone (ANOVA F(3,27) = 252.16, *p* = 0; Fig. 3d). The EMG response was not dependent on the stimulation order (Chi-squared test Χ^2^ (4, *N* = 28) = 5.5, *p* = .23; Bonferroni corrected pairs; AE–P, *p* = 0.19; AE-V, *p* = 0.45; P-V, *p *= 0.54; Fig. 3e).

Although motor responses in Fig. 3 and Supplementary Video 1 can appear unilateral, and in some cases bilateral, behavioural laterality cannot be taken as evidence of unilateral cortical target engagement under these conditions. At the ultrasound carrier frequency used here, 500 kHz, the acoustic wavelength in brain tissue is approximately 3 mm, which is a substantial fraction of the mouse head dimensions. Reflections at the skull and air boundaries can therefore generate standing waves and multiple intracranial pressure maxima. Finite-element simulations in a realistic mouse-head model showed a clear focal region under acoustically matched boundary conditions, but complex multi-maxima pressure distributions when boundary reflections were present, limiting spatial confinement in vivo (Supplementary Fig. S4).

These results suggest that applying an acoustic and electric field at the same frequency acoustoelectrically induces a DC electric field that is sufficiently strong to modulate neural activity.

### In vivo acoustoelectric AC neuromodulation

**Fig. 4:**
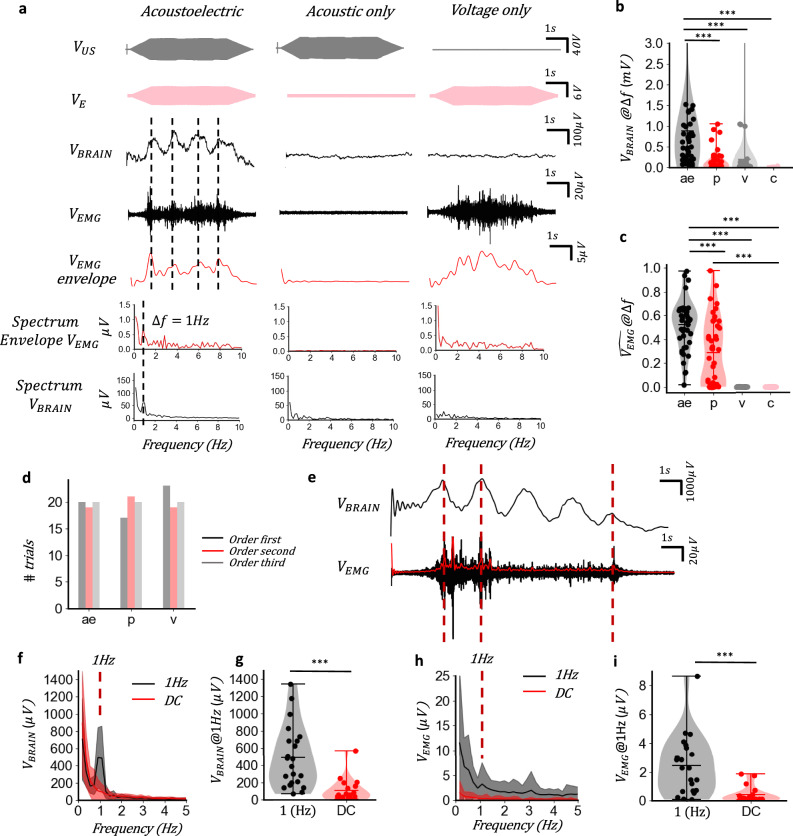
In vivo acoustoelectric neuromodulation @ Δ*f* = 1*Hz*. **a** Example counterbalanced triplet where the acoustic wave was applied at 500 kHz 1.8  MPa, and the bipolar voltage output stimulus at 12 Vpp, 500.001 kHz. $\Delta f=1{Hz}$ at ${V}_{{BRAIN}}$ and ${V}_{{EMG}}$. From spectrum, acoustoelectric = 510 $\mu V$, pressure = 80 $\mu V$, and voltage = 60$\mu V$. **b** Spectrum amplitude of ${V}_{{BRAIN}}$ at 1 Hz, calculated from the central 4 s of each recording to exclude onset and offset ramps. Data are presented as mean values ± s.d.; individual points represent triplet measurements, with *n* = 45 triplets from 9 mice. Groups were compared using one-way ANOVA followed by Tukey’s HSD post hoc test for pairwise comparisons: F(3,44) = 6.36, *p* = 0.0004, AE–P, *p* = 0.002; AE-V, *p* = 0.004; P-V, *p* = 0.5; AE-C, *p* = 0.02; P-C, *p* = 0.9; V-C, *p* = 0.9. **c** Spectrum amplitude of ${V}_{{EMG}}$ at 1 Hz compared using the log ratio of each triplet. Data are presented as mean values ± s.d.; individual points represent triplet measurements, with *n* = 44 triplets from 9 mice. Groups were compared using one-way ANOVA followed by Tukey’s HSD post hoc test for pairwise comparisons: F(3,43) = 370.69, *p* = 0.0, AE–P, *p* = 2.27e-5; AE-V, *p* = 0.00; P-V, *p* = 0.00; AE-C, *p* = 0.00; P-C, *p* = 0.00; V-C, *p* = 1.00. **d** Experimental order analysis. **e** Typical time series ${V}_{{BRAIN}}$ and ${V}_{{EMG}}$. **f** Frequency specificity comparison between ${V}_{{BRAIN}}$ at 1 Hz and 0 Hz, where ${V}_{{BRAIN}}$ has selection criteria such that the 1 Hz amplitude is larger than the 0.5 Hz amplitude, eliminating trials where electrochemical or pressure onset effects dominated, for *n* = 22 trials. ${V}_{{BRAIN}}$ comparison at $\Delta f$ = 0 and 1 Hz, with mean and s.d. error bar. **g** *T* test comparison between $\Delta f$ = 0,1 Hz acoustoelectric ${V}_{{BRAIN}}$ measurements, two sided t_(21)_  = 4.38, *p* = 8.5e-5; ${V}_{{BRAIN}}$ at 1 Hz $({\rm{mean}}\pm {\rm{s}}.{\rm{d}}.=491\pm 351{{\upmu }}{\rm{V}})$; ${V}_{{BRAIN}}$ at 0 Hz (${\rm{mean}}\pm {\rm{s}}.{\rm{d}}.=108.2\pm 130{{\upmu }}{\rm{V}}$). **h** ${V}_{{EMG}}$ comparison between 0,1 Hz datasets (**i**) *T* test comparison between $\Delta f=\mathrm{0,1}{Hz}$ acoustoelectric ${V}_{{EMG}}$, two sided t_(21)_ = 4.11, *p* = 0.00019; 1 Hz ${V}_{{EMG}}$, $({\rm{mean}}\pm {\rm{s}}.{\rm{d}}.=2.45\pm 2{{\upmu }}{\rm{V}})$; 0 Hz ${V}_{{EMG}}$, (${\rm{mean}}\pm {\rm{s}}.{\rm{d}}.=0.42\pm 0.55{{\upmu }}{\rm{V}}$). Source data are provided as a Source Data file.

We next tested neural responses to acoustic and electric fields applied at a difference frequency $\Delta f$ = 1 Hz (${f}_{{US}}$ = 500 kHz, ${f}_{E}$ = 500.001 kHz; *n* = 9 mice, 4-5 trials per mouse, 44 triplet trials total). Because EMG responses were only observable during a limited lightening-anaesthesia window, trials occurring prior to motor responsiveness were excluded, and variability in trial number reflects physiological state rather than experimental selection. During acoustoelectric stimulation, the 1 Hz difference frequency was present in both the brain-recorded signal and the envelope EMG but was absent when acoustic or electric fields were applied alone (Fig. 4a). Notably, the electric field alone (500.001 kHz) does not generate a difference frequency; the measurable EMG modulation arises from the low-frequency difference-frequency field ($\Delta f$ = 1 Hz) produced only when the acoustic and electric fields are applied simultaneously. We found that the 1 Hz component was most clearly expressed in the EMG when ultrasound pressure was reduced to minimise large DC onset and offset components, which otherwise dominated the low-frequency response. Accordingly, the data in Fig. 4 is presented to test the frequency specificity of acoustoelectric stimulation ($\Delta f$ = 1 Hz), rather than to provide a direct amplitude comparison with DC ($\Delta f$ = 0 Hz) conditions shown in Fig. 3. Because evoked responses varied strongly with anaesthesia depth, activation thresholds were not estimated from monotonic amplitude sweeps; instead, stimulation modalities were compared using counterbalanced, interleaved trials at matched amplitudes to assess relative response occurrence and amplitude. As in Fig. 3, behavioural laterality/extent was not used to infer spatial focality in vivo at 500 kHz, because intracranial standing-wave patterns can produce multiple pressure maxima (Supplementary Fig. S4).

To quantify the amplitude of the acoustoelectric signal at $\Delta f=1{Hz}$, we computed the spectrum using the Fourier transformation and extracted the amplitude at the 1 Hz bin. We found that the 1 Hz electric potential induced in the brain by the acoustoelectric stimulation was larger than those induced by the pressure or voltage alone, or a no stimulation control (ANOVA F(3,44) = 6.36, *p* = 0.0004; Fig. 4b). Similarly, the normalised EMG amplitude induced in the paw by the acoustoelectric stimulation was larger than those induced by the pressure or voltage alone (ANOVA F(3,43) = 370.69, *p* = 0; Fig. 4c). The EMG response was not dependent on the stimulation order (Chi-squared test Χ^2^ (2, *N* = 44) = 2.84, *p* = 0.24; Bonferroni corrected pairs AE–P, *P* = 0.83; AE-V, *P* = 0.27; P-V, *P* = 0.36; Fig. 4d).

At $\Delta f$ = 1 Hz, EMG responses were temporally sparse and non-sinusoidal, reflecting threshold-based activation rather than phase-locked entrainment, with physiological factors such as EMG habituation contributing to the observed response pattern^28^. DC components were occasionally observed, likely arising from a combination of standing acoustic waves in the mouse head (Supplementary Fig. S4), electrochemical offsets at the electrode interface (Supplementary Fig. S5), and onset responses to the acoustic field alone (Fig. 3b, pressure-only condition). In a typical signal (Fig. 4e), we found two time-correlated peaks at the beginning and one at the end. Shown in the illustrative example Supplementary Video 2, the variance in the acoustoelectrically generated amplitude in the measured brain signal ${V}_{{BRAIN}}$ can be seen, with the mouse responding via paw movements to only the largest voltage changes in the brain.

To validate that the 1 Hz spectral amplitude was not confounded by the DC offsets observed in some recordings, we repeated the analysis but with only the acoustoelectric recordings where the DC offset did not dominate the 1 Hz acoustoelectric signal. We found an evident peak at the 1 Hz difference frequency in both the electric signal recorded in the brain (Fig. 4f, g) and the paw EMG (Fig. 4h, i) that did not exist when the pressure and voltage were applied at the same frequency, i.e., DC acoustoelectric stimulation (amplitude spectral density (ASD) at 1 Hz vs 0 Hz: ${V}_{{BRAIN}}$: t(21) = 4.38, *P* = 8.5e-5; ${V}_{{EMG}}$: t(21) = 4.11, *P* = 0.00019). Together, these results demonstrate that the acoustoelectrically generated field in the brain governs the frequency content of the evoked neuromodulatory response, independent of acoustic or electrical stimulation alone.

### Acoustoelectric artefact tests

**Fig. 5:**
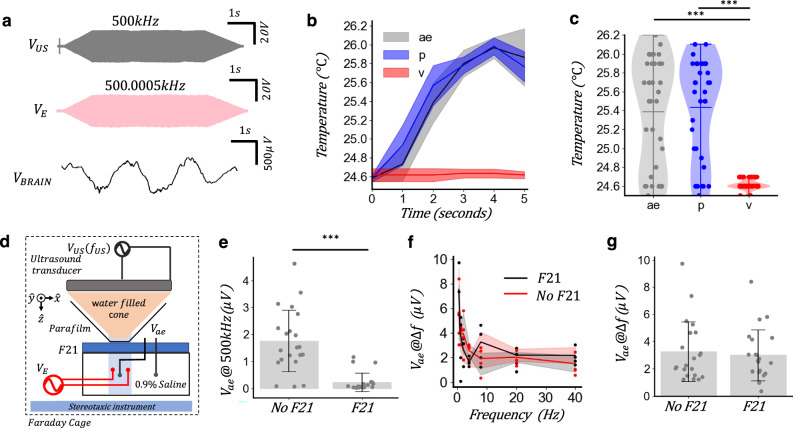
Acoustoelectric artefact tests. **a** Temperature measurement during acoustoelectric stimulation at Δ*f* = 0.5 Hz, with 1 MPa acoustic pressure and 40 Vpp applied voltage. A thermometer was placed in the ultrasound gel above the mouse skull and below the ultrasound cone; *n* = 6 repeat trials per acoustoelectric, pressure-only and voltage-only condition. ${V}_{{US}}$ at 500 kHz and 1 MPa, ${V}_{E}$ at 20 V, ${V}_{{BRAIN}}$ filtered below 10 Hz, revealing the 0.5 Hz acoustoelectric oscillation. **b** Temperature change from trial onset. Lines show mean values; shaded bands show ± s.d. **c** Temperature change across conditions. Data are mean ± s.d.; points are repeated post-baseline temperature measurements from 6 repeat trials per condition; n = 38 measurements total. One-way ANOVA with Tukey’s HSD: F(2,35) = 35.6, *p* = 1.5e-12; AE–P, *p* = 0.9; AE-V, *p* = 9e-10; P-V,* p* = 5.9e-11. **d** Saline petri-dish setup used to induce and measure an acoustoelectric field. **e** Electric field amplitude generated by the ultrasound transducer at 500 kHz, measured with and without F21. Data are mean ± s.d.; points are paired repeated measurements across 7 $\Delta f$ conditions with 3 repeats per condition; n = 21 paired measurements. Two-sided paired *t* test: ${{\rm{t}}}_{(20)}$ = 5.39, *p* = 0.00. Carrier amplitudes were measured after 5 kHz −3 dB low-pass filtering at the preamplifier front end. **f** Acoustoelectric $\Delta f$ amplitude as a function of $\Delta f$, with and without F21. Points show mean values; error bars show ± s.d.; *n* = 4 repeat measurements at each $\Delta f$ for each condition. **g** Acoustoelectric $\Delta f$ amplitude measured with and without F21 using an independently applied electric field. Data are mean ± s.d.; points are paired repeated measurements across 7 $\Delta f$ conditions with 3 repeats per condition; *n* = 21 paired measurements. Two-sided paired *t* test: ${{\rm{t}}}_{(20)}$ = 0.49, *p* = 0.63. Source data are provided as a Source Data file.

To determine if temperature elevation might be the cause of the response during acoustoelectric neuromodulation, we applied an acoustoelectric 0.5 Hz difference frequency where the acoustic field was 500 kHz and the applied voltage 500.0005 kHz (Fig. 5a). A temperature probe was placed in the ultrasound gel above the skull and below the end of the ultrasound cone while a continuous 6 second acoustoelectric signal was applied, repeating 6 times for pressure or electric field alone, and electric and pressure field together to generate an acoustoelectric difference frequency at 0.5 Hz. While applying continuous ultrasound at 1 MPa does increase the temperature (Δ*T* ≈ 1.5 °C, Fig. 5b, shown in blue), the acoustoelectric and pressure only trials had a very high mean temperature correlation (0.98; Pearson, Fig. 5b), and no significant group difference (AE-P = 0.9, *n* = 36; Fig. 5c). This suggests the pressure and acoustoelectric trials are not differentiated by temperature, unlike the EMG and brain signal differences in the acoustoelectric versus pressure only experiments shown in Fig. 4. Hence, temperature is unlikely to be the cause of acoustoelectric neuromodulation.

To eliminate the electric field only mixing between the transducer artefact electric field (${V}_{{US}}$) and the independently applied electric field (${V}_{E}$) as the cause of the difference frequency, we calibrated an acoustoelectric field in a saline petri-dish to have the focal maximum at the measurement electrodes (Fig. 5d, with calibration procedure and spatial map described in Supplementary Fig. S6). The electric signal powering the ultrasound created an electric artefact at the pressure frequency, which could be measured in the saline petri dish and attenuated by inserting electrically insulating F21 material (Fig. 5e; *p* < 0.001). An acoustoelectric field was measured with and without the electrically attenuating F21 material, at a series of electric field difference frequencies to induce a spectrum (Fig. 5f). There was no difference in the acoustoelectric amplitudes between the electrically attenuating F21 and no F21 group (Fig. 5g; *p* = 0.63), suggesting that the induced acoustoelectric signal did not originate from mixing with the electric field artefact from the ultrasound transducer.

Non-linearity in the recording hardware or stimulus-generation path could also generate an electric difference frequency. To test whether the recording chain could produce this artefact, we fed the driving voltage signals for the electric and acoustic stimulation to the input of the recording preamplifier via an isolation transformer. No signal was detected at the difference frequency, suggesting that the acoustoelectric signal did not originate from frequency mixing in the recording hardware. We also monitored the pressure-drive and voltage-stimulus outputs during the Δf = 1 Hz in vivo experiments. The 1 Hz component was present in the brain-recorded signal but not in the monitored stimulation outputs, indicating that the difference frequency did not originate from electrical mixing in the signal-generation path (Supplementary Fig. S7).

### In vivo transcranial ultrasound stimulation is impacted by the electrical artefact powering the transducer

Now that we have evidence that acoustoelectric neuromodulation is possible when an independent electric field and a propagating acoustic field are applied to an ionic medium, we investigated the impact of the electric field artefact generated by powering the ultrasound transducer, to determine if it may also interact with the propagating acoustic field in ultrasound stimulation.

To determine the origin of the electric signal present in the mouse brain at the acoustic frequency, we first placed an acoustically transparent and electrically insulating material (2 mm thick F21, Precision Acoustics, UK; characterised in Supplementary Fig. S2) between the end of the ultrasound cone and the mouse. We found the electric signal at the acoustic frequency was attenuated (Supplementary Fig. S8a–c). We then electrically isolated the transducer from ground by inserting a switch into the cable connecting the transducer to the RF amplifier, which massively increased the amplitude of the electric signal transmitted into the mouse (Supplementary Fig. S8d–f) as the energy injected into the transducer needed to find a path to ground via nearby ionic media such as the mouse. These results suggest the electrical artefact from powering the transducer capacitively transmits electrical energy into nearby ionic media, similar to an electrical antenna^29^.

Using a saline phantom, we next tested whether the transducer-driven electrical artefact could undergo acoustoelectric mixing with the propagating acoustic field. Pulsed ultrasound alone, delivered at 500 kHz with a 1 kHz pulse-repetition frequency, generated electrical components at the acoustic carrier and at low-frequency envelope components measured by electrodes in the saline. A simple simulation of the pulsed carrier reproduced the expected baseband components generated by multiplication of the ultrasound-drive waveform with its pressure envelope (Supplementary Fig. S9). Spatial mapping showed that the low-frequency and sum-frequency components were focal with the acoustic field, whereas the carrier-frequency artefact was not. Inserting 2 mm F21 between the ultrasound cone and saline attenuated the focal sum- and difference-frequency components as well as the spatially broad carrier artefact. These results indicate that the transducer-driven electrical artefact can interact with the propagating acoustic field to generate acoustoelectric components, rather than arising from electrical mixing alone or acoustic radiation force alone (Supplementary Fig. S10).

**Fig. 6:**
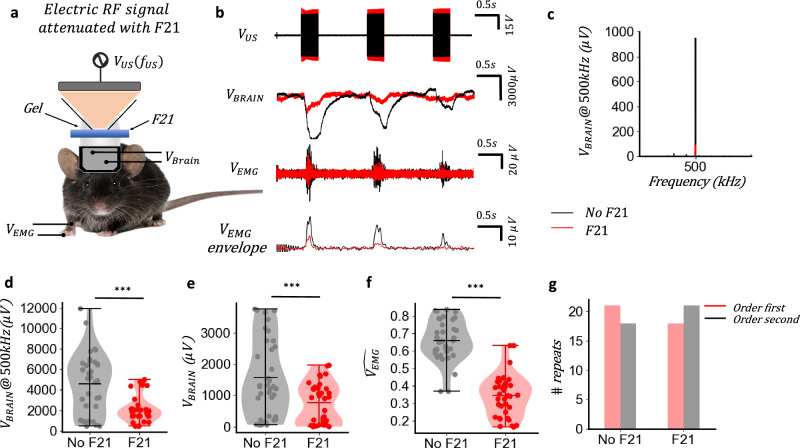
In vivo ultrasound brain stimulation with attenuation of the electrical artefact from the ultrasound transducer. **a** Mouse undergoing pulsed ultrasound stimulation with F21 electrical attenuation material in place. Mouse photograph licensed from Shutterstock, image ID 160253894, ©Michiel de Wit/Shutterstock, used under Standard License. **b** Representative with (red) and without (black) F21 experiment, with ${V}_{{US}}$ shown 1 kHz low pass filtered at ${V}_{{BRAIN}}$, ${V}_{{EMG}}$ and envelope ${V}_{{EMG}}$. Pressure without F21 = 2 MPa; pressure with F21 = 2.4 MPa. **c**, Representative amplitude spectrum at ${V}_{{BRAIN}}$ comparison between the electric artefact with and without F21. **d** carrier amplitude comparison; *t* test, two sided ${t}_{(35)}$ = 4.65, *p* = 1.47e-5; F21 group $({\rm{mean}}\pm {\rm{s}}.{\rm{d}}.=2895\pm 1253\mu V)$; without F21 group (${\rm{mean}}\pm {\rm{s}}.{\rm{d}}.=4601\pm 2116\mu V$) *n* = 7 mice, 36 trials, during light ketamine/xylazine anaesthesia. **e** ${V}_{{BRAIN}}$ comparison log normalised to take pair based pressure differences into account; *t* test, two sided ${t}_{(35)}$ = 3.52, *p* = 7e-4; F21 group (mean ± s.d. = 755 ± 630$\mu {\rm{V}}$) No F21 group (mean ± s.d. = 1572 ± 1217*μV*) using data as in (**d**). **f** ${V}_{{EMG}}$ normalized per pair to account for changing anaesthesia; *t* test, two sided on log normalized pair; ${t}_{\left(35\right)}=4.65$, *p* = 1.4e-5; F21 group $({\rm{mean}}\pm {\rm{s}}.{\rm{d}}.=0.34\pm 0.11\mu V);$ No F21 group (${\rm{mean}}\pm {\rm{s}}.{\rm{d}}.=0.665\pm 0.11\mu V$) using data as in (**d**). **g** Experimental order analysis. Source data are provided as a Source Data file.

To decouple the ultrasound acoustic field and electric artefact in vivo, we performed an F21 electrical shielding experiment (Fig. 6a) with a pulsed ultrasound stimulation protocol where the pulse was on 0.5 s and off for 1.5 s (25% duty cycle) at 500 kHz, as these parameters have been reported to be highly effective at eliciting an evoked ultrasound stimulation response previously^30^.

During the lightening anaesthesia window, a 2 mm F21 layer was inserted into the gel below the ultrasound cone to reduce the transducer-drive electrical artefact recorded at the intracranial electrodes. The insertion of the 2 mm F21 layer produced a small, reproducible attenuation of acoustic pressure (Supplementary Fig. S2b); therefore, in all experiments, the transducer drive ${V}_{{US}}$ was increased by 15% when F21 was present (Fig. 6b), ensuring that delivered acoustic pressure in the brain was matched across F21 and no-F21 conditions. The F21 layer was then removed and the procedure repeated 1–2 times per mouse, alternating trial order to minimise anaesthesia depth confounds (Supplementary Fig. S3).

In Fig. 6b we show a typical representative example, where we applied a pulsed ultrasound signal without F21 and saw it produced a larger amplitude low- frequency brain signal than the group with F21 in place at 2.4 MPa. The resulting EMG signal measured without F21 has larger spikes, while electrically shielding the transducer with F21 yielded smaller EMG responses (shown in red in Fig. 6b, shown with simultaneous video and measurements in Supplementary Video 3). EMG responses also reduced on the third pulse as compared to the first pulse, and the low-frequency electrical signal measured in the brain (${V}_{{BRAIN}}$) was not always the same amplitude per pulse, despite the pressure output being the same amplitude for each pulse in a trial.

Analysis of the spectral amplitude of the transducer-induced electrical artefact (${V}_{{US}}$) (Fig. 6c) shows that F21 selectively attenuates the electric carrier component measured in the brain. At the group level, with 7 mice yielding a total of 36 pulses with F21/no F21 pairings, the electrical insulation properties of F21 attenuate the electrical carrier artefact from the transducer (Fig. 6d, p = 1.47e-5). This trend is mirrored in the pair-normalised brain signal amplitude comparison (Fig. 6e, *p* = 7e-4), as well as the EMG signal amplitudes (Fig. 6f, *p* = 1.4e-5). Across paired trials, attenuation of the carrier artefact with F21 was accompanied by a reduction in both the low-frequency brain response (${V}_{{BRAIN}}$) and the evoked EMG amplitude, consistent with an acoustoelectric contribution under these stimulation conditions.

Finally, to ensure that the order of trials was not responsible for the increased amplitude when no F21 was present we performed a Chi-squared test on the counterbalanced data showing there was no significant relationship between the order of trials and the amplitude of the EMG response Χ^2^ (3, *N* = 35) = 1.32, *p* = 0.24 (Fig. 6g). Hence, in vivo ultrasound-evoked responses can be attenuated by reducing the amplitude of the transducer-induced electric field in the medium, independent of acoustic pressure and time-dependent anaesthesia depth changes.

### In vivo ultrasound stimulation is not due to electric field only mixing

**Fig. 7:**
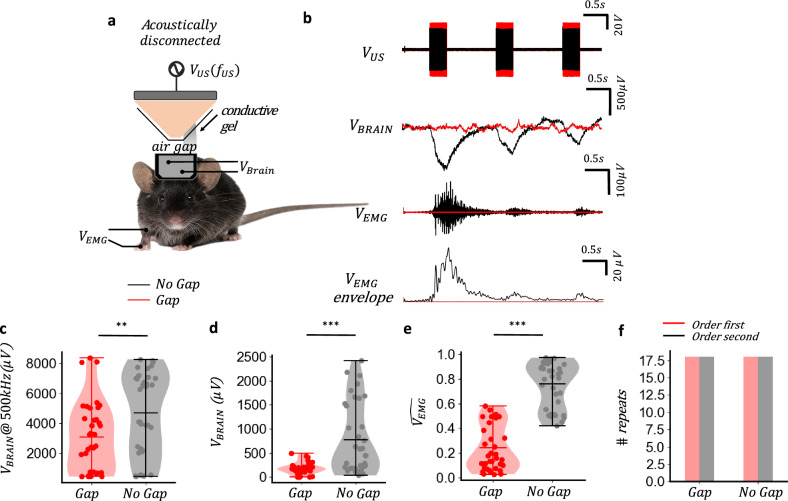
Ultrasound stimulation is not due to electric mixing. **a** Instrumentation arrangement for acoustically disconnected measurements in the mouse brain, in which the acoustic wave was blocked by an air gap while the transducer remains electrically connected to the brain via conductive gel. Mouse photograph licensed from Shutterstock, image ID 160253894, ©Michiel de Wit/Shutterstock, used under Standard License. Recordings were 6 seconds long and used 0.5 s, 500 kHz, 1 MPa acoustic pulses. **b** RF output signal for 1 MPa pressure, showing that the acoustically connected amplitude (black) was 20% lower than acoustically disconnected amplitude, low frequency brain signal ${V}_{{BRAIN}}$, (< 1 kHz) comparing no gap/acoustically connected stimulation (black) against electrical connection only/air gap stimulation (red), EMG trace ${V}_{{EMG}}$ filtered between 100 Hz and 1 kHz for both no gap (black) and gap (red) conditions and Hilbert transformed EMG traces (< 10 Hz) for no gap and gap conditions. **c** Carrier amplitude measured via the brain electrodes for gap (red) and no gap (black) conditions. Data are mean ± s.d.; individual points represent pulse measurements from *n* = 6 mice, with 2 trials per condition and 3 pulses per trial, yielding *n* = 36 pulses per condition. Groups were compared using a two-sided paired *t* test: ${t}_{(35)}$  = −3.23, *p *= 0.0018; gap $({\rm{mean}}\pm {\rm{s}}.{\rm{d}}.=3066\pm 2302\mu V);$ no gap (${\rm{mean}}\pm {\rm{s}}.{\rm{d}}.=5967\pm 4773\mu V$). **d** Brain-signal height comparison between gap and no-gap conditions. Data are mean ± s.d.; individual points represent pulse measurements from *n* = 6 mice, yielding *n* = 36 pulses per condition. Groups were compared using a two-sided paired *t* test: ${t}_{(35)}$ = -4.79, *p* = 8.81e-6; gap, 174.2$\pm 117.2\mu {V;}$ no gap 771.2$\pm 726.9\mu V$. **e** EMG comparison between gap and no-gap conditions across n = 6 mice, with 2–3 trials per mouse. EMG amplitudes were normalised within each trial to account for time-dependent variation, and statistics were performed on log-normalised values. Data are mean ± s.d.; individual points represent normalised pulse measurements, yielding n = 36 paired pulse measurements. Groups were compared using a two-sided paired *t* test: ${{\rm{t}}}_{(35)}$ = -9.06, *P* = 2e-13; gap, 0$.24\pm 0.18$; no gap, 0$.75\pm 0.18$. **f** Order analysis. Groups were compared using a chi-squared test: χ²(3, N = 35) = 0.16, *p* = 0.68. Source data are provided as a Source Data file.

To determine if the stimulation effect may be due to the electric field artefact from the 500 kHz electric field applied by the ultrasound transducer mixing with itself at the measurement electrodes, we performed an acoustic disconnection test where the electric field remained connected via gel connection with the ultrasound cone, but the acoustic field was removed by leaving an air gap below the end of the cone (Fig. 7a). To overcome a slight reduction in the amplitude of the electric field when a smaller volume of conductive gel was present, we added 15% to the voltage driving the ultrasound transducer.

The representative plots (Fig. 7b) show the voltage recorded at the measurement electrodes in the brain (${V}_{{BRAIN}}$), where no EMG was evoked in any trial in the electric artefact only case, while the acoustic field did induce a response (Fig. 7b, and Supplementary Video 4). This was repeated across 6 mice, with 2 trials of each case recorded for each mouse, each with 3 pulses recorded. The carrier frequency measured in the brain was similar between acoustically connected (no gap) and disconnected (gap) groups, though the larger surface area gel connection enabled better electrical transmission (Fig. 7c; *p* = 0.0018). There were no DC offsets in the brain when there was an air gap blocking the acoustic signal (Fig. 7d, *p* = 8.8e-6), and no EMG responses were evoked (Fig. 7e, *p* = 6.0e-18). The difference between the measurement with and without the air gap was not affected by the measurement order (Fig. 7f).

These results support the evidence that the DC electric field present in the brain tissue is generated by an acoustoelectric interaction between the ultrasound and its electric artefact.

## Discussion

Non-invasive electrical brain stimulation shows promise as an alternative to invasive neurosurgical approaches for treating epilepsy^31^, obsessive compulsive disorder^32^ (OCD), depression^33^, Alzheimer’s disease^34^, and for inducing neurogenesis after stroke^35^, while avoiding the risks of surgical intervention. However, clinical translation requires techniques that deliver better targeting, controllability and safety. Ultrasound stimulation offers high spatial specificity and depth, yet uncertainty about its mechanism persists^36,37^. A modality that harnesses the well‑characterised electrical models of neural signalling while retaining the spatial focality of ultrasound would enable independent optimisation of amplitude and safety through acoustic and electric control axes.

Acoustoelectric neuromodulation achieves this via a non-linear interaction between an applied electric field and a focused ultrasound field, generating a focal difference-frequency electric field that can drive neuromodulation. Using the peak intracranial acoustoelectric potential observed in vivo ( ~ 9 mV at onset; Fig. 3b) and the spatial scale from phantom maps ( ~ 1.5 mm between extrema at 500 kHz; Fig. 2b, c), we estimate a focal low-frequency field of order ~ 6 V/m, corresponding to an induced current density $J \sim \sigma E\approx 1-4$ $A/{m}^{2}$ for $\sigma \approx 0.2-0.6S/m$ (order-of-magnitude; geometry dependent). The observed amplitude and frequency dependence of responses on the acoustoelectrically generated difference frequency argues against pressure‑only mechanisms as the primary driver. For example, acoustic radiation force^38^ (ARF) can be dissociated from the difference frequency response by varying the applied electric-field frequency: when a 1 Hz difference frequency is generated and evokes stimulation at 1 Hz, ARF alone cannot account for the effect because the response also requires the independently applied high‑frequency electric field. Taken together, the results argue against pressure-only accounts - including non-linear acoustics^39^, acoustically induced thermal dissipation^9,40^, cavitation^41^, or auditory pathways^10,42^ - as sufficient explanations.

Acoustoelectric neuromodulation is also not explained by electric mixing only from the transducer drive and an independent applied electric field. The artefactual electric field produced by powering the transducer is not directly proportional to the acoustoelectric difference‑frequency signal, based on testing with an electrically attenuating material. Pressure‑induced vibration of recording electrodes, which could generate non‑linear electrochemical artefacts^43^, is likewise unlikely: free‑field saline phantom experiments showed that acoustoelectric amplitude depends on the geometry of the applied fields in the medium^17^, inconsistent with a simple vibration artefact.

Because the ultrasound drive voltage can capacitively couple into surrounding ionic media, an artefactual electric field coexists with the propagating acoustic field during ultrasound stimulation. Since both occur at the same frequency, their interaction yields a difference frequency at 0 Hz (DC). The dependence of response amplitude on the electric‑field magnitude present in the medium when acoustic and electric frequencies are matched (Fig. 3) indicates that acoustic‑only mechanisms - non-linear acoustics^39^, thermal dissipation^9,40^, cavitation^41^, and auditory pathways^10,42^ - cannot solely account for ultrasound‑evoked neuronal activation. Conversely, electric‑field–only mixing produced by the transducer drive did not elicit stimulation, arguing against purely electrical or electrochemical origins. Together, these observations indicate that both acoustic and electric fields are required to generate a focal difference‑frequency electric field and that the acoustoelectric interaction makes a substantial contribution to ultrasound brain stimulation.

Although acoustoelectric neuromodulation is motivated by the potential for spatially confined electric-field generation, the present in vivo mouse experiments do not demonstrate localised cortical stimulation, and the observed motor responses reflect physical constraints of the model rather than limitations of the underlying mechanism. At the ultrasound carrier frequency used here, the relationship between mouse head size and acoustic wavelength promotes non-focal standing waves (Supplementary Fig. S4). Using higher‑frequency transducers or larger heads (e.g., humans) should reduce standing‑wave artefacts and yield more focal acoustoelectric fields^36^. In addition, direct neural spiking analyses (e.g., post-stimulus time histograms or spike–stimulus coherence) would require stable single-unit or multi-unit recordings under conditions not compatible with the present acute stimulation paradigm and are therefore left to future studies. We note that the continuous-wave exposure used here (TI ≈ 1.4; ${{\rm{I}}}_{{\rm{spta}}}={{\rm{I}}}_{{\rm{sppa}}}\approx 32.5{\rm{W}}/{{\rm{cm}}}^{2}$) lies within the ITRUSST safety guidelines for focused ultrasound stimulation^44^. Accordingly, translation of this approach to human applications will require waveform optimisation and careful consideration of skull-induced heating, attenuation and aberration^45^.

Acoustoelectric neuromodulation offers advantages over ultrasound alone. It leverages the extensive literature on how electric fields influence neurons^23^ and allows rapid in vitro optimisation of focused electrical parameters. With acoustic and electric inputs as independent control axes, safety and efficacy can be tuned separately.

Future work should further isolate and quantify the respective acoustic and electric contributions to ultrasound-evoked responses in vivo. These responses may depend on the electrical environment, including grounding, transducer geometry, and the electric field necessary to drive the transducer^46^; endogenous electric fields may also shape the net field inside neural tissue. More broadly, acoustoelectric interactions are likely to influence related modalities that combine acoustic and electromagnetic fields, including magneto‑acoustic neuromodulation with static fields^47^, MR‑guided tFUS^36^, and conventional ultrasound brain stimulation.

Our results demonstrate that acoustoelectric mixing can directly elicit neuromodulation and constitutes a key mechanism underlying ultrasound‑induced brain stimulation. By clarifying how acoustic and electric fields interact within neural tissue, this work advances spatially precise, non‑invasive control of brain activity.

## Methods

### In vivo mouse experiment

#### Animals

Experiments were performed in wild-type C57BL/6 J mice, male and female, aged 3–6 months. Both male and female mice were included; sex was not used as a biological variable in the statistical analysis because the study was designed to test the physical requirement for simultaneous acoustic and electric fields rather than sex-specific differences, and sample sizes were not powered for sex-disaggregated comparisons. Mice were obtained from Charles River Laboratories. Mice were housed in standard cages in Imperial College London animal facility, with ad libitum food and water in a controlled light-dark cycle environment, with standard monitoring by veterinary staff. The Imperial College of London’s Animal Welfare and Ethical Review Body approved all animal procedures under project licence PPL P2EA80855, and all experiments were performed in accordance with relevant regulations/according to the United Kingdom Animals (Scientific Procedures) Act 1986.

#### Surgery

Mice were anaesthetised using 1-3% (vol/vol) isoflurane in oxygen and subcutaneously administered Vetergesic (0.1 mg/kg) and Carprofen (5 mg/kg) to inhibit pain and inflammation, and saline for hydration (10 ml/kg/hour). A Neurotar compatible head plate was attached to the mouse using clear dental cement (PalaXpress Dental Cement, AgnTho’s AB, Sweden) and a craniotomy performed with a drill (diameter 0.5 mm) at the intended electrophysiological measurement sites. The position of the craniotomy relative to the bregma was anteroposterior 0.0 mm, mediolateral − 2 mm for the motor cortex electrode and anteroposterior − 3.5 mm, mediolateral 2.25 mm for the visual cortex reference electrode used to measure electrical amplitudes within the motor cortex. The recording electrodes were 98% platinum-iridium wire segments (0.5 mm diameter wire, VWR, Lutterworth, UK) that were positioned over the drill holes so that the end of the wire was touching the brain, and dental cement secured it to the head bar. Nail polish was then applied to the electrodes to electrically insulate the platinum-iridium wire, so that only the portion of the wire located in the brain was exposed. Low-toxicity silicone sealant (Kwik-Cast, World Precision Instruments) was then poured around the electrodes to seal moisture around the craniotomy site and over the exposed skull, whilst also stabilising the electrodes in position. The mouse was then recovered in a warming chamber and administered Carprofen (5 mg/kg) for three days after surgery with daily weight cheques, then allowed to recover for one week before experiments commenced.

#### Ultrasound fields application

The acoustic field was applied using a curved ceramic (PZT) 500 kHz ultrasound transducer (60 mm diameter, 63.5 mm acoustic path length; Precision Acoustics Ltd, UK). The ultrasound transducer was driven by an arbitrary function generator (Handyscope HS5, TiePie engineering, Netherlands) and a 40 W linear power amplifier (240 L, Electronics & Innovation Ltd). The ultrasound transducer was mounted in a cone and filled with water characterised in Supplementary Fig. S1. Non-conductive Parafilm was stretched over the end of the cone to seal the water in, while the transducer was positioned via stereotaxic instrument above the mouse head. F21 was inserted in the gel to attenuate the electrical signal in the mouse brain emanating from the ultrasound transducer. Ultrasound gel (Anagel, UK) was applied on top of the mouse head, surrounding all electrodes, and providing an acoustic connection between the end of the ultrasound cone and the top of the mouse skull.

#### Electric fields application

The electric field was applied using a pair of 98% platinum-iridium wire electrodes (Alfa Aesar, 0.25 mm diameter), that were formed into loops to increase the exposed surface area, further decreasing electrochemical induced offsets when the bipolar electric field was applied. One loop was gently inserted into the mouth (loop diameter 1 cm), and the second located above the head (loop diameter 2 cm), placed between the ultrasound cone and the mouse head within the acoustic gel interface. The electrodes were driven by an arbitrary function generator (WiFiScope WS5, TiePie engineering, Netherlands) and a custom-made high-frequency voltage source made of glass core transformers (Hitachi Metals) with a maximum ±20 V amplitude at an effective 3 dB bandwidth range of 5 kHz to 2 MHz. To output voltages which surpassed the 12 V function generator range, a 20 W linear power amplifier was used (325LA, Electronics & Innovation Ltd). The transformer’s isolation prevented extraneous tissue charging and minimised total harmonic distortion.

#### Electrophysiology recordings

The electrode implant was connected to an SR560 preamplifier (Stanford Research Systems, USA) via a 1.27 mm Dupont header, which was cemented onto the back of the head bar. A low-pass filter (5 kHz -3dB frequency with circuit diagram in Supplementary Fig. S12) was added whenever a high-frequency electric field was applied to prevent overloading the preamplifier.

#### Electromyography (EMG) recordings

EMG was measured using stainless steel EMG electrodes (0.3 mm diameter, Arbitter, UK) that were placed in the front paw, with the second electrode at the muscle innervation point on the forearm. The EMG electrodes were then connected to a differential amplifier (SR560, Stanford Research Systems) with a band pass filter (100-1 kHz) to isolate just the EMG signal.

#### Data acquisition

All applied and monitored signals were recorded at 5MS/s, and were time synced using a combined multiple instrument (CMI) interface between the function generators and oscilloscopes with trigger inputs. The applied voltages, monitor channels and electric potentials were logged using data acquisition hardware (WiFiScope WS5, WiFiScope WS6 DIFF and Handyscope HS5, TiePie engineering, Netherlands). The logged signals were streamed from the data acquisition hardware to a workstation PC using custom C code, which utilised the Tiepie SDK to control the triggers, channels, and function generator output. The compiled C code, which called the oscilloscope commands, was controlled through a Python wrapper script. An instrumentation diagram and connection details available in Supplementary Fig. S11. The low-pass filter described in Supplementary Fig. S12, was used in only the measurements with a large amplitude independently applied electric field (Fig. 5 and below).

#### Experimental procedure

Anaesthesia was induced via subcutaneous injection of ketamine (75 mg/kg) and xylazine (10 mg/kg). Eye lubricant (Vaseline) was placed on the eyes to prevent them from drying. The anaesthetised mouse was placed onto a thermal mat (Thermostar, RWD, China) to maintain homoeostasis at 37 °C and head position stabilised with the installed head clamp (Neurotar standard clamp, Finland) enabling precise stereotaxic localisation of the ultrasound transducer over the head. The intracranial recording electrodes were connected to the recording amplifier, and the EMG electrodes were placed in the front paw and forearm and connected to the recording amplifier. Ultrasound gel (Anagel, UK) was applied on top of the mouse head, surrounding all electrodes to provide an acoustic connection between the end of the ultrasound cone and the top of the mouse skull. The stimulating loop electrodes were placed above the head and in the mouth as described above. The US transducer was positioned above the mouse head using a stereotaxic instrument. A F21 material was inserted into the gel to attenuate the US electrical artefact.

Acoustic and electric fields were then applied according to the instrumentation description (see Supplementary Fig. S11 and S12). Each experimental block included the three conditions (i.e., E, US, E + US), applied for 6 seconds with a 0.25-second ramp up and down periods in a counterbalanced order.

EMG responses could only be elicited during the period in which anaesthesia depth lightened, typically occurring approximately 45–60 min after ketamine/xylazine administration. Prior to this window, no EMG activity was observed regardless of stimulation amplitude. Beginning approximately 30 min after anaesthesia induction, a 1 MPa acoustic stimulus was applied at 5 min intervals to monitor the transition into the lightening anaesthesia window. Experiments commenced once evoked EMG responses were observed and were terminated once spontaneous movements began. Because EMG responsiveness changed as ketamine/xylazine anaesthesia lightened, stimulation conditions were counterbalanced within triplets to reduce anaesthesia-depth confounds. Mice were then administered atipamezole (Antisedan; 1 mg/kg, subcutaneous) to reverse xylazine and transferred to a warming chamber for recovery. Following recovery, animals were provided with water-saturated food pellets and returned to their home cage.

Pressure amplitude ranged between 0.5-2.5 MPa. Typical in vivo rodent experiments performed in tFUS experiments have largely been done when the mouse is fully awake^30,48–50^, requiring lower pressure thresholds to evoke EMG and often with spontaneous movements also present. Here, experimental measurements were performed when the anaesthesia was light, and there were no spontaneous movements which required pressure magnitudes that were slightly higher than those measured in awake mice.

#### Temperature recording experiments

Temperature measurements were performed using a temperature probe positioned in the ultrasound coupling gel directly above the skull and immediately below the distal end of the ultrasound cone. A continuous acoustic signal was applied for 6 s and repeated six times, while temperature was recorded continuously. Ultrasound was delivered in continuous-wave (CW) mode at 500 kHz with a spatial-peak pressure amplitude of 1 MPa at the focus, as measured in water using a calibrated hydrophone (Supplementary Fig. S1). For a sinusoidal acoustic plane progressive wave, the spatial-peak intensity ($I$) is given by: $I=\frac{{p}_{{pk}}^{2}}{2\rho c}$, where ${p}_{{pk}}$ is the peak pressure, $\rho$ is the medium density, and $c$ is the speed of sound. Using $\rho=1000{kg}/{m}^{3}$ and $c=1540m/s$, this corresponds to a spatial-peak pulse-average intensity of ${I}_{{sppa}}\approx 32.5W/{{cm}}^{2}$. Since stimulation was continuous (duty cycle = 1), the spatial-peak temporal-average intensity was, ${I}_{{spta}}={I}_{{sppa}}\approx 32.5W/{{cm}}^{2}$, yielding a thermal index of approximately 1.4.

### Phantom measurements

#### Petri dish phantom

A small plastic petri dish (4 cm diameter and 2 cm depth) filled with 0.9% saline located where the mouse would be positioned within the Faraday cage used for in vivo electrophysiology experiments.

#### Tank phantom

A custom-built acrylic tank (50 cm × 20 cm × 20 cm) filled with 0.9% saline. An acoustic damping material (Aptflex-48) was attached to the back of the tank to minimise acoustic reflections. The phantom tank was covered with a Faraday cage.

#### US fields application

Similar to the in vivo experiment.

#### Electric fields application

Similar to the in vivo electric field application, but with the wire electrode forming short pins placed with a 10 mm spacing around one of the recording electrodes.

#### Electrical recording

Similar to the in vivo intracranial electric field recording, with the electrode implant used for measurement and the same spacing as the in vivo experiments.

#### Pressure recordings

The acoustic field was measured in the tank phantom using a 0.2 mm needle hydrophone with a calibration of 52 mV/kPa at 500 kHz provided by the manufacturer, Precision Acoustics Ltd and a DC-coupled preamplifier (Precision Acoustics Ltd).

#### Data acquisition

Similar to the in vivo data acquisition.

#### Procedure to identify US focus using the AE effect

To locate the acoustic focus in a saline phantom, an electric field was applied at 8 kHz and the ultrasound transducer moved in 0.5 mm increments to find the peak acoustoelectric amplitude at 492 kHz. The acoustoelectric mixing amplitudes reached a maximum when the ultrasound focus was positioned over the voltage stimulation source (see Supplementray Fig. S6). In the saline phantom, detailed calibration maps could be made like the ones shown (Supplementary Fig. S6b).

#### Spatial measurement procedure

For the 2-dimensional acoustoelectric free field maps recorded in a biomimicking tank phantom (20 cm x 20 cm x 50 cms) filled with 0.9% saline, a recording was made for each pixel consisting of a ramped electric field at the beginning and end to avoid voltage spike transients in the preamplifier. To time sync individual recordings to one another, a half-amplitude 2 wavelength duration acoustic field was applied at the beginning of the signal. Once all measurements were made, each file was time aligned to the embedded acoustic field marker, after filtering to isolate the sum and difference frequencies, the individual files were extracted into a large three-dimensional array including time and two dimensions. This enabled the recovery of ${\phi }_{{ae}}$ $\left(t\right),$ such that we can obtain the time evolution of the generated $\hat{x}\hat{y}$ and $\hat{x}\hat{z}$ views.

### Signal processing and data analysis

Signal processing and data analysis were performed using custom Python (v3.13.7) scripts that utilised functions from the NumPy (v2.2.6), SciPy (v1.16.3), and Pandas (v2.3.3) Python libraries.

### Computation of EMG envelope

The EMG envelope was computed by performing a Hilbert transform and extracting the amplitude of the resulting analytical signal. A low-pass filter with a cutoff frequency of 10 Hz was applied to the resulting envelope.

### Frequency domain analysis

A 1-D discrete Fourier Transform with a Flat Top window to optimise amplitude accuracy was computed on the central 4 s of each recording, as we report amplitude spectral density (ASD) instead of power spectral density (PSD) to easily read the average amplitude of each measurement. After the Fourier transform is computed, the two-sided amplitude spectrum is multiplied by 2, and half the array is taken, converting it into its single-sided form.

### EMG amplitude normalisation

The EMG amplitude (i.e., ASD at $\Delta f$) at each stimulation condition was normalised to the total amplitude across the conditions at each triplet block to account for inter-block variation in responsiveness due to, for example, changes in anaesthetic level. Statistical tests were performed on the log values.

### Peak alignment analysis

Peaks in the brain electric potential signal and EMG envelope signal were identified using the Python-based find_peaks function. An alignment between the peaks was defined as occurring when the EMG peak coincided with a peak of the brain signal, provided the time difference was within 1/5 of the period (i.e., 0.2 seconds for 1 Hz stimulation).

### Statistics and reproducibility

No statistical method was used to predetermine sample size. Sample sizes were determined by the feasibility of the in vivo mouse preparation, the limited lightening-anaesthesia window in which EMG responses could be evoked, and the number of successful counterbalanced trials obtained per animal. Animals were not randomly assigned to separate intervention groups because stimulation conditions were tested within-subject. Stimulation conditions were applied in counterbalanced order, where indicated, to reduce confounding by anaesthesia-depth drift. Investigators were blinded to the counterbalanced condition order during acquisition and outcome assessment; condition labels were assigned after analysis. Blinding was not possible for the hardware setup and stimulation preparation.

Data were excluded from statistical analyses only when anaesthesia was too deep to evoke any EMG response despite the presence of the Δf frequency in the brain, as described in Supplementary Fig. S3. All files were otherwise included in the dataset, including recordings with poor signal quality or electrochemical offsets, unless a panel-specific criterion is explicitly stated. For the Fig. 4 frequency-specificity analysis, recordings were included when the 1 Hz brain-signal amplitude exceeded the 0.5 Hz amplitude to reduce contamination by electrochemical or pressure-onset effects.

Statistical analyses were performed in Python using NumPy, SciPy and Pandas. Unless otherwise stated, data are presented as mean values ± s.d. Statistical tests, sidedness, degrees of freedom, exact *P*-values, *n*-values and definitions of the experimental unit are provided in the figure legends. For comparisons involving counterbalanced triplets, EMG amplitudes were normalised within triplets to account for changes in anaesthesia depth, and statistical tests were performed on log-transformed normalised values where stated.

### Reporting summary

Further information on research design is available in the Nature Portfolio Reporting Summary linked to this article.

## Supplementary information

Supplementary Information

Description of Additional Supplementary Files

Supplementary Video 1

Supplementary Video 2

Supplementary Video 3

Supplementary Video 4

Reporting Summary

Transparent Peer Review file

## Source data

Source Data

## Data Availability

The data supporting the findings of this study are available within the paper, its Supplementary Information and the Source Data file. The time-series and analysed datasets have been deposited in Figshare under DOI: 10.6084/m9.figshare.c.7909283. Source data are provided in this paper.

## Code availability

Code supporting the findings in this article is available at https://github.com/Acoustoelectric/Acoustoelectric_neuromodulation_and_its_contribution_to_US_stimulation, with 10.6084/m9.figshare.c.7909283.
