# Supplementary material for: Non-invasive in vivo acoustoelectric neuromodulation and its contribution to ultrasound stimulation
Source: Nat Commun. 2026 Jun 17;17:5073. doi: 10.1038/s41467-026-73826-2 (PMC13276402; doi:10.1038/s41467-026-73826-2)
Supplement: Supplementary file 1 — Supplementary Information [file 41467_2026_73826_MOESM1_ESM.pdf]

Supplementary Information: Non-invasive in vivo acoustoelectric neuromodulation and its contribution to ultrasound stimulation

Jean L. Rintoul[1*], Christopher Butler[2], Robin O. Cleveland[3], Nir Grossman[1*]

[1]Department of Brain Sciences, Imperial College London, London, UK. [2]The George Institute for Global Health, School of Public Health, Imperial College London, London, UK. [3]Institute of Biomedical Engineering, University of Oxford, Oxford, UK

*Corresponding authors: j.rintoul19@alumni.imperial.ac.uk; nirg@imperial.ac.uk

SUPPLEMENTARY INFORMATION

1. Acoustoelectric and acoustic field characterization

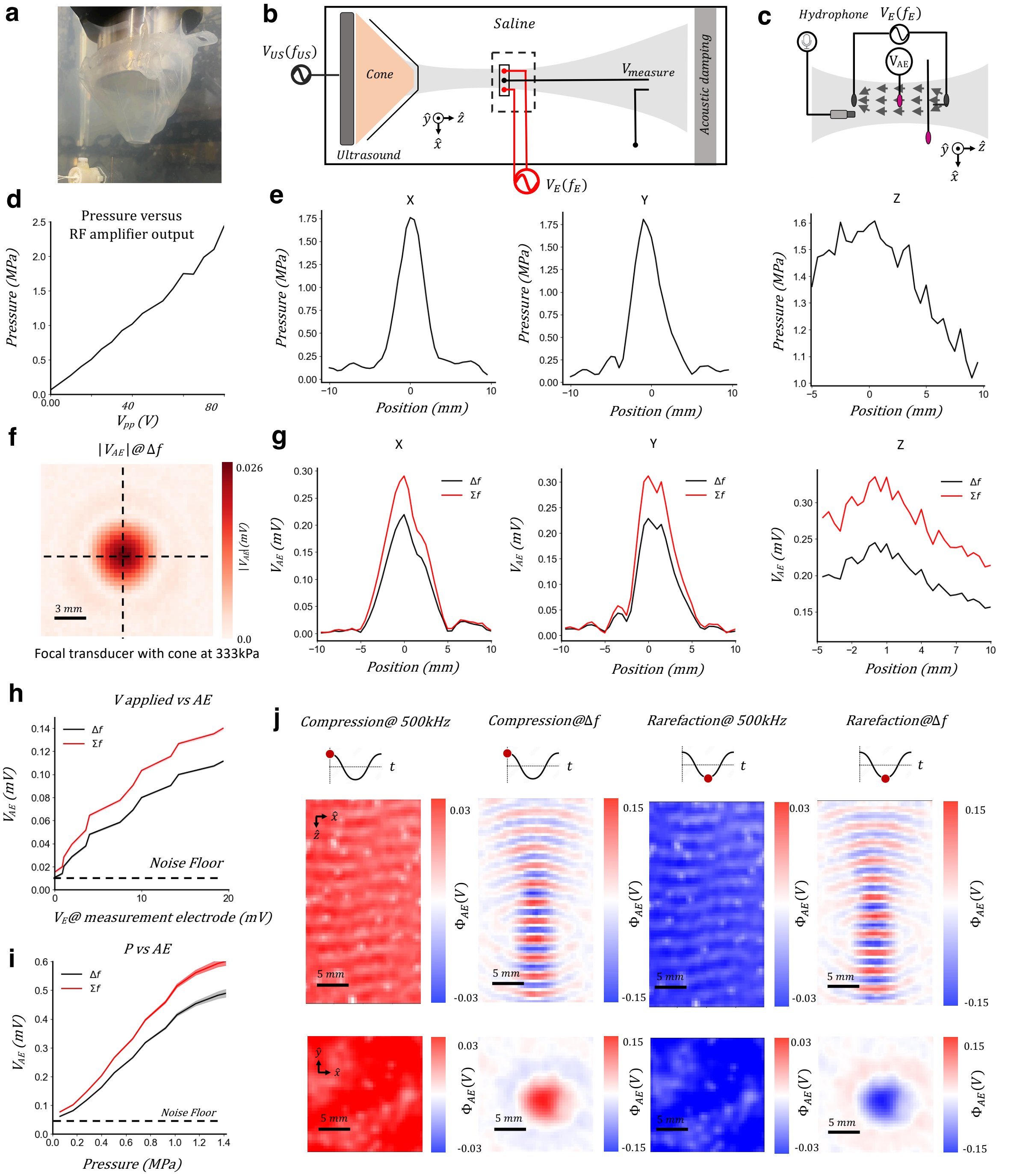

Figure S1 | Acoustic and acoustoelectric cone characterization a, 500kHz ultrasound transducer with cone (70mm diameter at the top, 30mm at the bottom, 50mm height), covered in parafilm. **b,** Phantom tank for XYZ scanning of electric fields generated through the acoustic focal area. **c,** hydrophone and electrical measurement electrodes for measuring acoustoelectric effect. **d,** Output voltage of the RF amplifier versus output pressure measured at the focal spot of the ultrasound transducer in 0.9% saline with hydrophone. **e,** XYZ mapping of the acoustic amplitude measured with a hydrophone. Z depth axis is made irregular by reflections off the back of the tank, despite the use of acoustic damping material. **f,** XY spatial distribution map of acoustoelectric difference frequency generated with 500kHz pressure at 333kPa, and 20Vpp@8kHz on voltage electrodes aligned with the ultrasound. 0.1s duration at 5MHz sampling rate, for each pixel. Data band filtered around difference frequency of 492kHz. **g,** Similar parameters to f, except showing X, Y and Z axis sum (508kHz) and

difference (492kHz) frequencies and their distribution for each axis. **h,** Ramping amplitude of applied 8kHz voltage with 500kHz pressure constant amplitude at 0.4MPa. Shown is sum and difference voltage amplitudes calibrated to the focal point of the transducer. 8kHz was chosen, so that a shorter time length of recording could be used in each measurement compared to an electric field at a similar frequency to the acoustic signal. **i,** Ramping pressure of 500kHz ultrasound transducer with constant 12V output 8kHz voltage on electrodes. Shown are the sum and difference voltage amplitudes calibrated to the focal point of the transducer. **j,** XY and XZ spatial maps of acoustoelectric difference frequency and acoustic carrier frequency for both a compression and rarefaction. Source data are provided as a Source Data file.

2. F21 material characterization

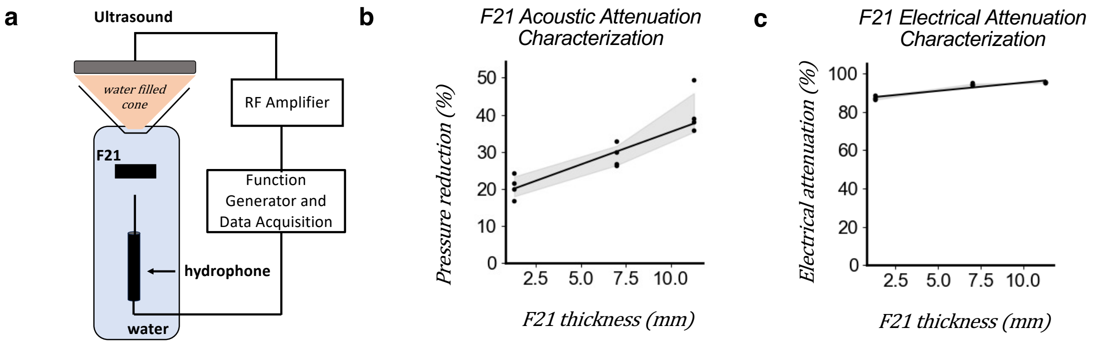

Figure S2 | F21 acoustic and electrical characterization a, Hydrophone and transducer arrangement for F21 characterization. **b**, F21 material acoustic attenuation versus thickness. 8 second duration recordings were chosen to mimic the continuous field applied in experiments, 5MHz sampling rate repeated 4 times at each F21 thickness. Regression line: Attenuation (%) = 1.75 * thickness (mm) + 17.8 **c**, F21 material electrical attenuation versus thickness. Regression line: Attenuation (%) = 0.86*F21 thickness (mm) + 86. Source data are provided as a Source Data file.

3. Impact of changing anaesthesia depth

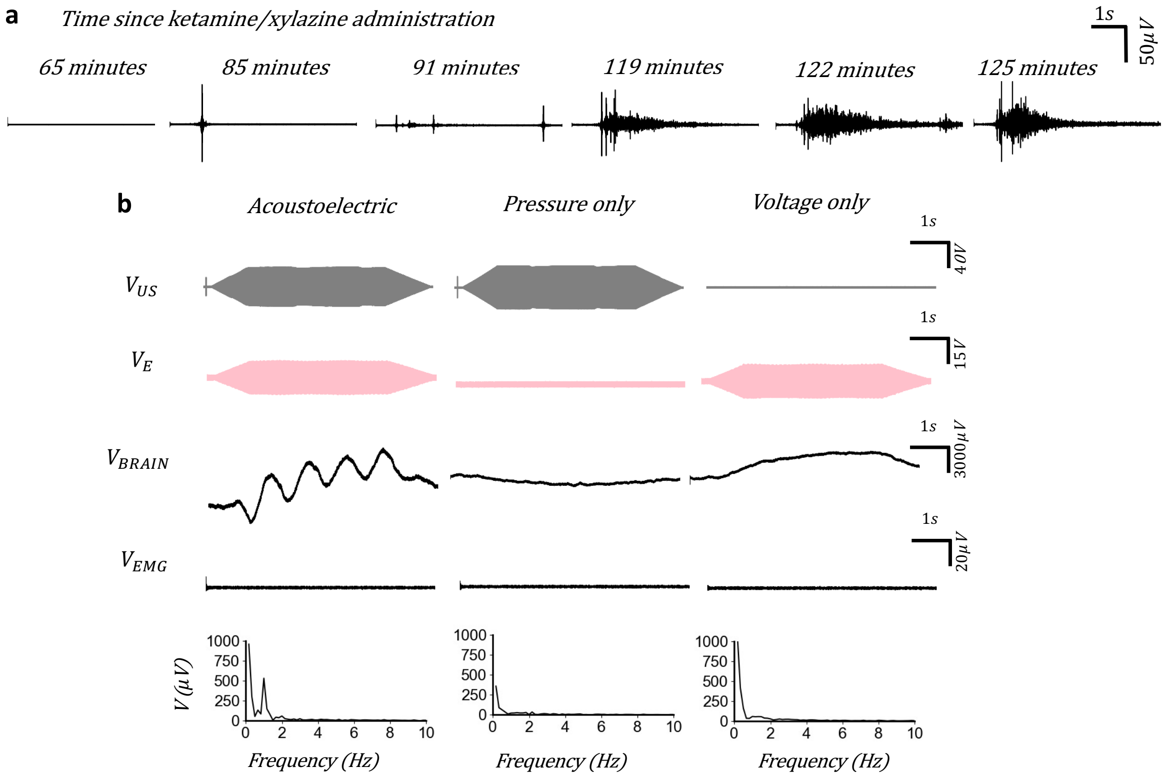

Figure S3 | Impact of changing anaesthesia depth. a, Time series showing EMG trace change when ultrasound stimulation is applied at increasing time intervals since the beginning of ketamine/xylazine administration. EMG filtered between 100-1kHz, acoustic signal kept constant in all representative plots (2MPa, 500kHz), sampling rate 5MHz, duration 10s. **b,** Data exclusion example due to depth of anaesthesia; Δf=1Hz, P=2MPa, V = 30Vpp, Brain signal amplitudes: acoustoelectric signal = 3.396$mV\ pp$, pressure only = 1$mV\ pp$, voltage only = 3$mV\ pp$**.** Leave one out experiment example performed when anaesthesia was too deep to evoke any EMG response, despite Δf=1hz being present at sufficiently high amplitudes in the brain.

4. Standing waves in the mouse head

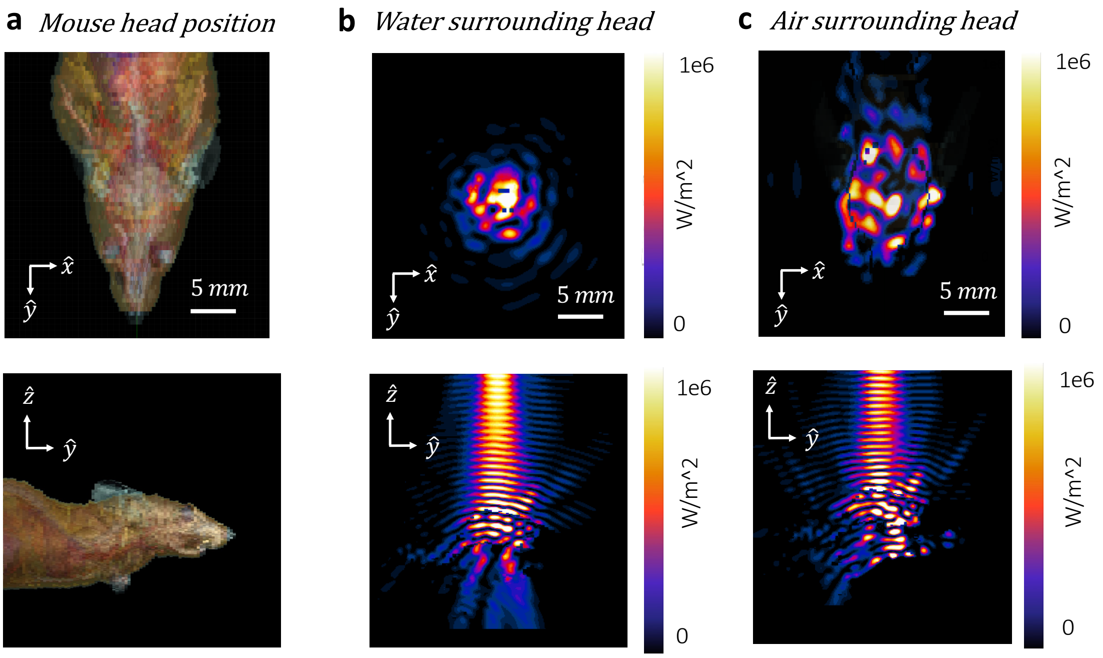

Figure S4 | Acoustic standing wave reflections. a, Geometric inhomogeneous mouse model used in Sim4Life finite element modelling simulations with 500kHz ultrasound transducer matching our physical transducer experiments. **b,** 100 acoustic cycles simulated geometry in a mouse surrounded by water. The focal area is clearly in the middle. **c,** 100 acoustic cycles simulated geometry in a with mouse surrounded by air. The acoustic impedance boundary induces emergent standing waves, diminishing the achievable focality at these length scales.

5. Example of poor data included in the statistics: Electrochemical effects at high voltages

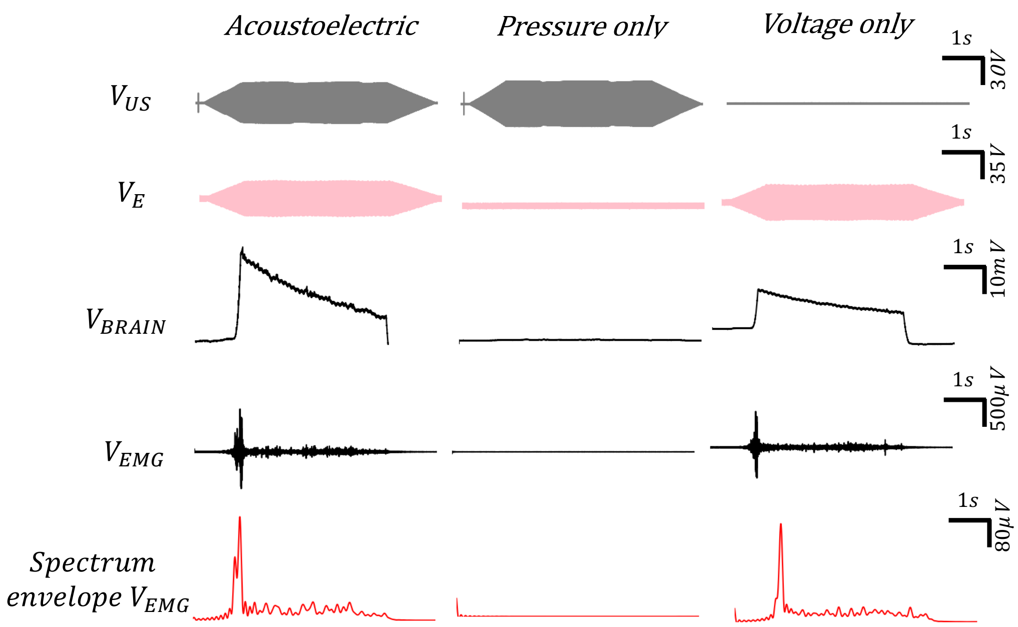

Figure S5 | Poor data: electrochemical DC offset effects at high voltages. $\Delta f = 1hz$ P = 1.5MPa, V = 70Vpp. Brain signal amplitudes: acoustoelectric signal = 22.15$mV\ pp$, pressure only = 1.5$mV\ pp$, voltage only = 19.9$mV\ pp$. At very high voltages non-linear electrochemical effects at the electrode interface obfuscate the acoustoelectric difference frequency in the brain signal, presenting as a large DC offset due to the charge imbalance between the stimulation electrodes.

6. Acoustoelectric position calibration technique

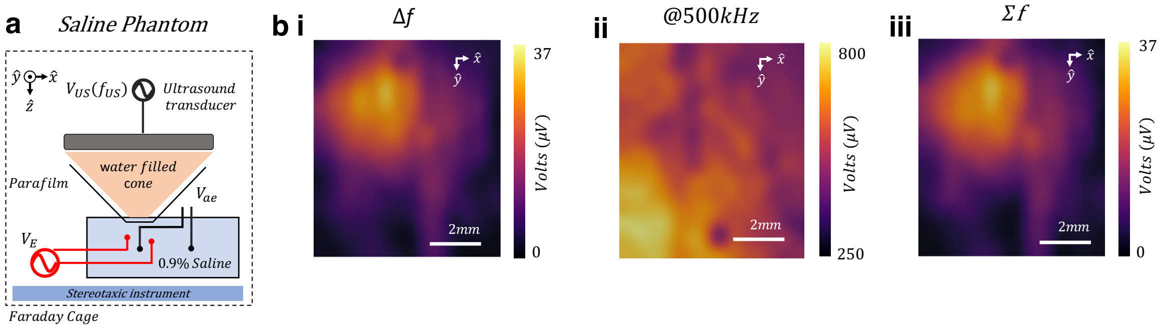

Figure S6 | Acoustoelectric position calibration. a, Stimulation and measurement arrangement for position calibration in a saline phantom Note: this method didn't work well in a mouse due to more complex reflections and standing waves and limited experimental timeline. **b,** Spatial calibration of stimulation electrode located at visual cortex with 1MPa 500kHz acoustic wave, and 1V output at 8kHz electrical signal on stimulation electrodes directly from function generator, using manual stereotaxic movement to recreate spatial image. i) Δf amplitudes over a 1cm square showing maxima at focus location. ii) Carrier frequency amplitude over same space has no focality. iii) Σf amplitudes over a 1cm square showing maxima at focus location.

7. Electric frequency mixing is not significant in the signal generation path

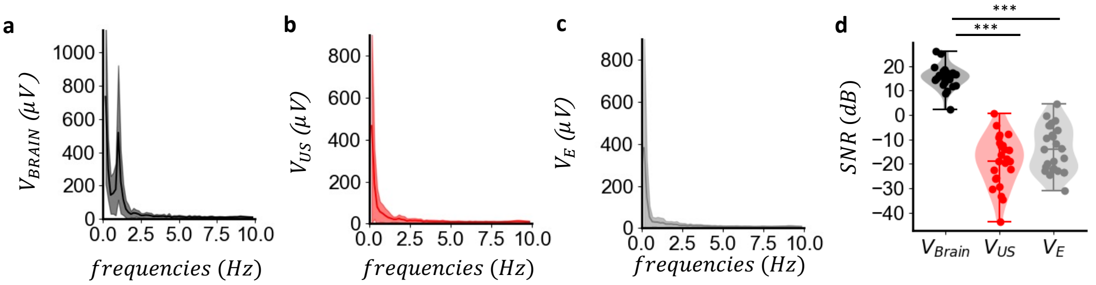

Figure S7 | In vivo signal-generation frequency-mixing test. a, In vivo measurement of V_{Brain} amplitude spectral density (ASD) from the same Δf = 1 Hz in vivo acoustoelectric dataset shown in **Fig. 4. b**, Monitored RF amplifier output, V_{US}, ASD from the same dataset**. c,** Monitored voltage-stimulus output, V_E, ASD from the same dataset. **d,** Signal-to-noise ratio (SNR) at 1 Hz, with noise defined as the mean amplitude between 0 and 2 Hz. Data are presented as mean values ± s.d.; V_{Brain} was consistently above 0 dB, whereas V_{US} and V_E were below 0 dB. Groups were compared using two-sided paired t-tests: V_{Brain} versus V_{US}, $t_{(21)}$= 15, p = 9.5e-20; V_{Brain} versus VE, $t_{(21)}$ = 14, p = 1.3e-18. Mean ± s.d. SNR values were 15.37 ± 4.7 dB for V_{BRAIN}, −18.81 ± 10.11 dB for V_{US}, and −14.10 ± 9.16 dB for V_E. Source data are provided as a Source Data file.

8. Identifying the source of the RF electrical artefact at 500kHz

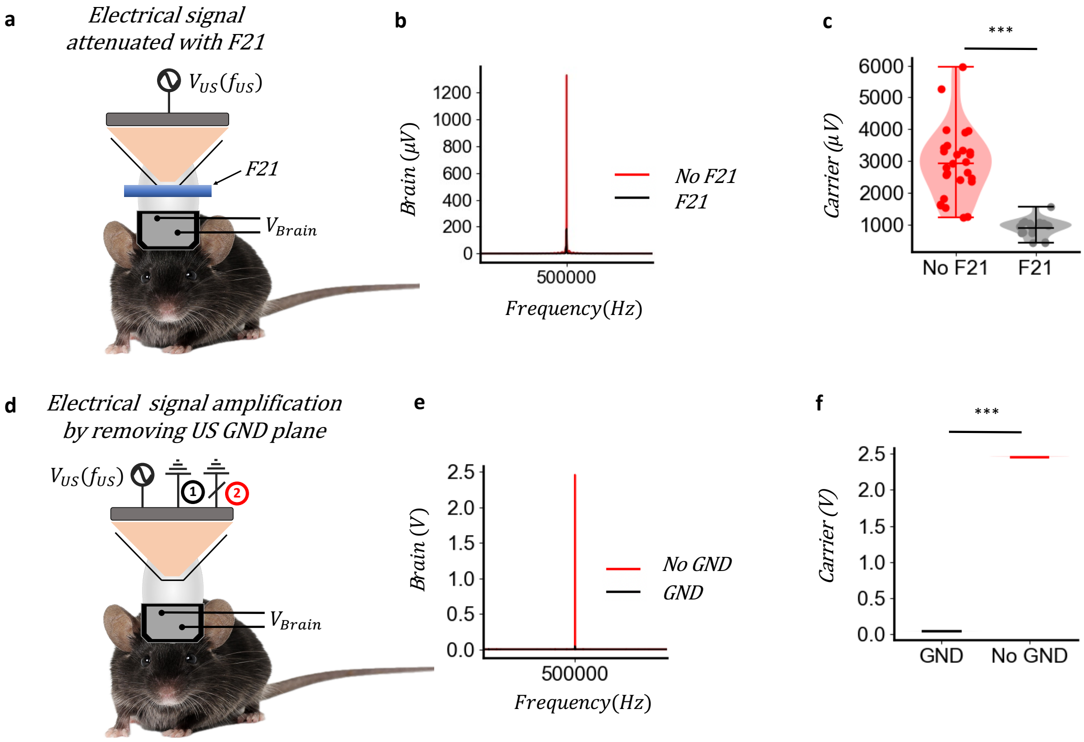

Figure S8 | Identifying the source of the electrical artefact. a, In vivo arrangement used to test electrical attenuation by F21 while preserving acoustic coupling. A 2 mm F21 layer was placed between the ultrasound cone and the mouse. Mouse photograph licensed from Shutterstock, image ID 160253894, ©Michiel de Wit/Shutterstock, used under Standard License. **b**, Representative amplitude spectral density (ASD) of the acoustic-carrier electrical artefact measured via the brain electrodes with and without F21. **c**, Carrier amplitude with and without F21 in place. Data are mean ± s.d.; No F21: n = 27 measurements from 9 recordings; F21: n = 18 measurements from 6 recordings; 5 mice. Two-sided independent-samples t-test: $t(43) = 7.52$, $p = 2.26 \times 10^{-9}$. Source data are provided as a Source Data file. **d**, In vivo arrangement used to test electrical artefact amplification by disconnecting the ultrasound transducer ground plane using a switch installed in the cable. Mouse photograph licensed from Shutterstock, image ID 160253894, ©Michiel de Wit/Shutterstock, used under Standard License. **e**, Representative ASD showing increased 500 kHz electrical artefact when the ground plane was disconnected. **f**, Carrier-amplitude comparison with the ultrasound transducer ground connected and disconnected. Data are presented as mean values ± s.d.; individual points represent paired repeated measurements. Groups were compared using a two-sided paired t-test: $t(4) = -1207.06$, $p = 1.47e-22$; no ground, 2.46 ± 3.90 mV; ground, 0.04 ± 0.40 mV. Source data are provided as a Source Data file.

9. Simulation of V_{US} compared with V_{US} x P_{US}

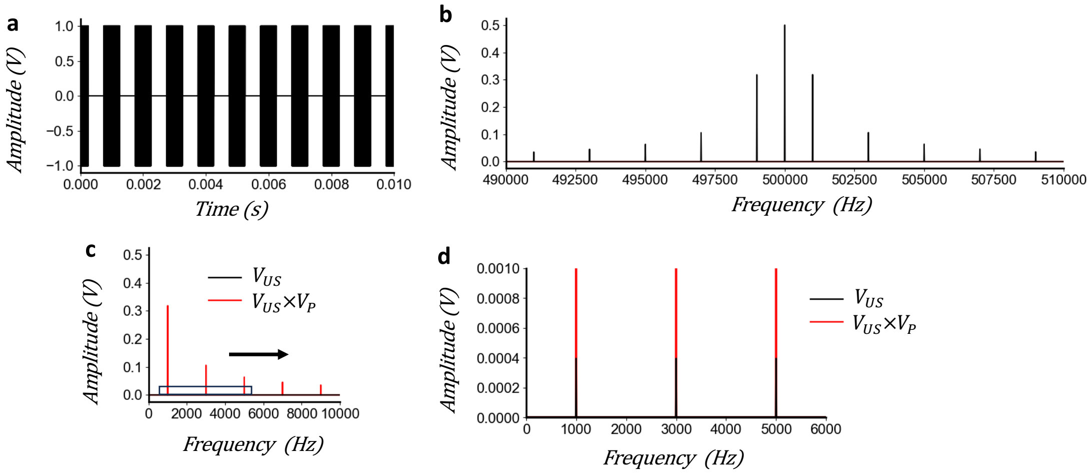

Figure S9 | Simulation of V_{US} compared with V_{US} x P_{US}. a, V_{US} simulated 500kHz sinusoid with amplitude 1, pulsed at 1kHz. **b,** ASD of V_{US} around the carrier. **c,** ASD at low frequencies comparing V_{US} and V_{US} x P_{US} signal. **d,** Zoom view around the first 3 frequencies, showing residue small V_{US} signals all at similar amplitudes and larger V_{US} x P_{US} signals decreasing in amplitude, mirroring the V_{US} spectrum shape except base banded around 0hz.

10. Ultrasound induces an electric field at the sum and difference frequency

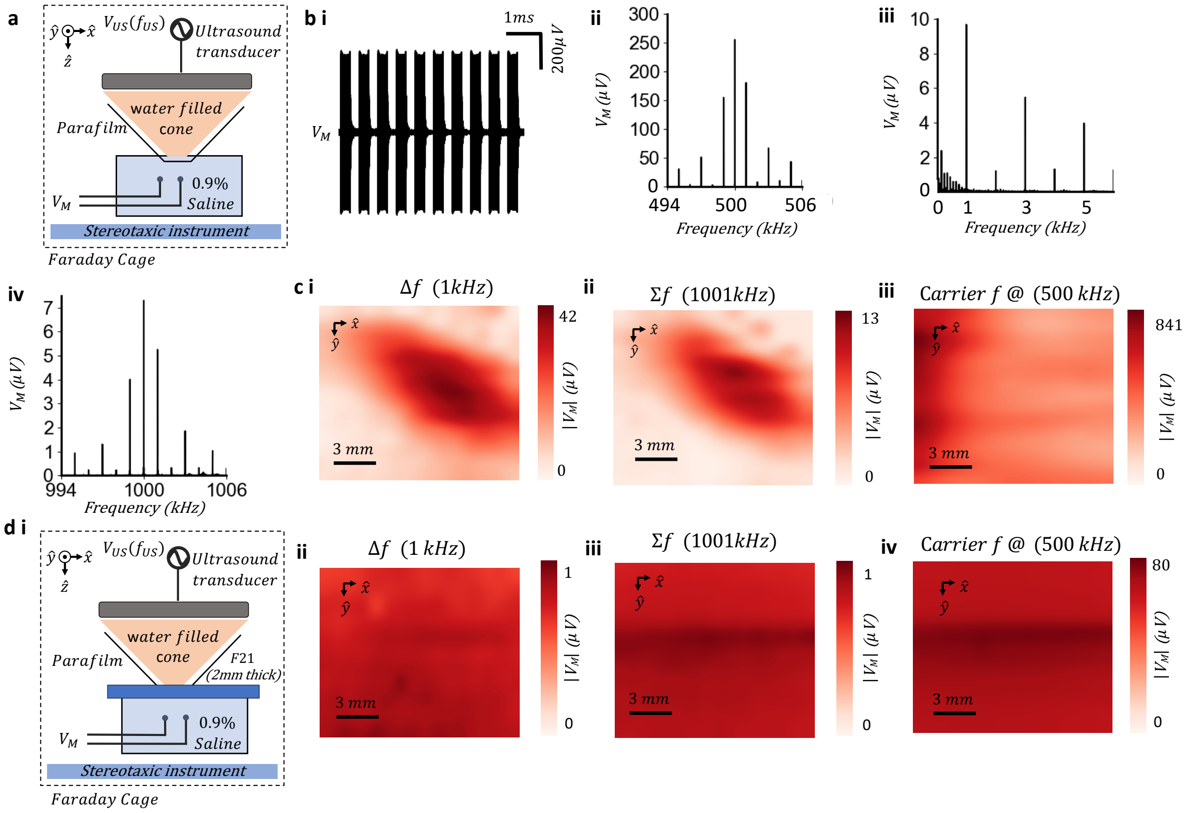

Figure S10 | Ultrasound undergoes an acoustoelectric interaction induced by the transducer electrical artefact. a, *In vitro* phantom arrangement in a petri dish filled with 0.9% saline. Pulsed ultrasound was applied at 500 kHz with a pulse-repetition frequency (PRF) of 1 kHz, 50% duty cycle and 1 MPa peak pressure. Platinum-iridium measurement electrodes were spaced 7 mm apart, and the stereotaxic instrument was moved in 0.5 mm increments to generate spatial maps. **b,** Representative

electrical measurement from the saline phantom. i), Time-series measurement at V_M showing the 1 kHz PRF waveform. ii), Amplitude spectral density (ASD) around the 500 kHz acoustic carrier. iii), Low-frequency ASD around the difference-frequency/envelope components. iv), High-frequency ASD around the sum-frequency components. **c,** Spatial maps without F21. i), Difference-frequency map. ii), Sum-frequency map. iii), Carrier-frequency map. **d,** Phantom measurements as in c, with 2 mm F21 placed between the saline petri dish and the end of the ultrasound cone. i), Experimental arrangement with F21. ii), Difference-frequency map. iii), Sum-frequency map. iv), Carrier-frequency map. Source data are provided as a Source Data file.

11. Acoustoelectric Neuromodulation Instrumentation

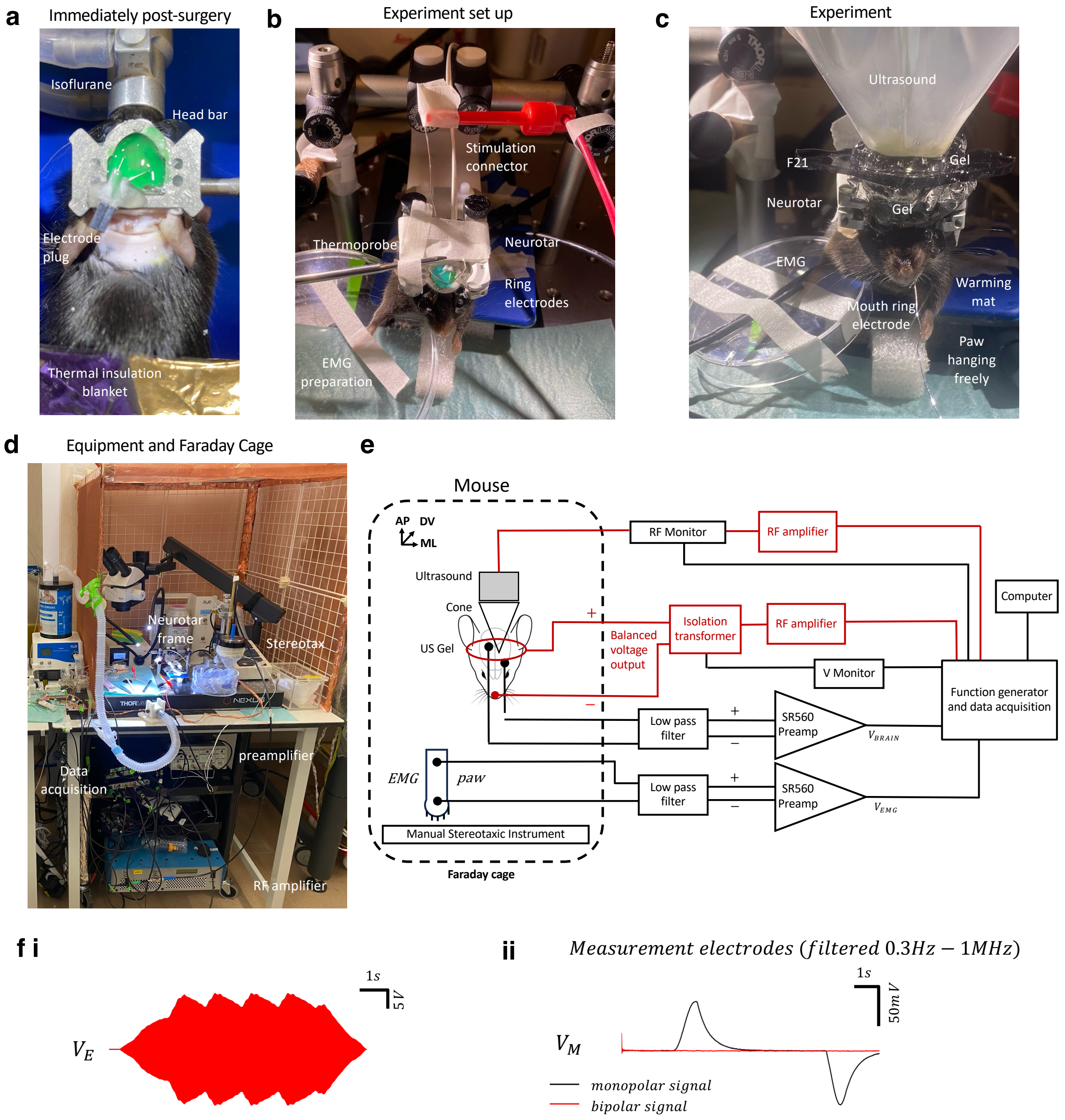

Figure S11 | Acoustoelectric instrumentation. a, Electrodes implant and head bar installation immediately post-surgery **b,** Experiment preparation with stimulation ring electrode arrangement, and EMG preparation under Ketamine/Xylazine anaesthesia. **c,** Mouse during experiment with EMG measurement, ultrasound cone, gel and 2mm F21 in place. **d,** Photo of faraday cage showing Neurotar frame for mouse and hardware equipment below. **e,** Acoustoelectric neuromodulation instrumentation block diagram for acoustoelectric neuromodulation. Red lines show stimulation equipment and black

lines show measurement system. **f,** i) Stimulation signal monitored during 70V peak-to-peak 500kHz voltage signal applied via the Platinum Iridium electrodes used for stimulation in the mouse, some reverberation is present due to electric reflections. ii) Differential measurement at electrodes in 0.9% physiological saline phantom, sampling rate 5Mhz, with differential preamplifier front end filter 0.3Hz-1MHz, gain=1, 12 seconds duration showing monopolar arrangement DC offset at onset and offset (black line) and bipolar signal (red line) where both have been 40Hz software low-pass filtered to reveal the electrochemical DC drift induced between the stimulation electrodes. Note that the preamplifier front end filter was implemented (0.3Hz high pass) to keep the recorded signal within its saturation limits, meaning the recorded filtered signal shows the onset of the DC but not the actual offset.

12. Low pass filter

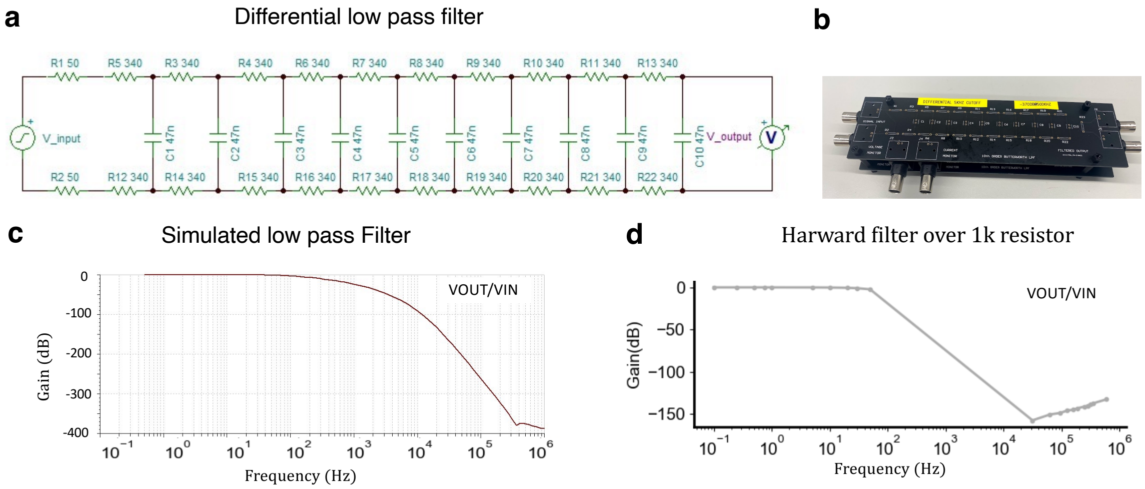

Figure S12 | Low pass filter. a, Circuit for low pass differential filter attenuating 300dB at 500kHz (based on pSPICE simulation). **b,** low pass filter was required to filter out the large amplitude high frequency electric signal delivered around 500kHz. **c,** simulated transfer function up to 1MHz. **d,** Real transfer function of the physical hardware filter.
