# Supplementary material for: Non-invasive in vivo acoustoelectric neuromodulation and its contribution to ultrasound stimulation
Source: Nat Commun. 2026 Jun 17;17:5073. doi: 10.1038/s41467-026-73826-2 (PMC13276402; doi:10.1038/s41467-026-73826-2)
Supplement: Supplementary file 2 — Description of Additional Supplementary Files [file 41467_2026_73826_MOESM2_ESM.pdf]

Description of Additional Supplementary Files

Supplementary Video 1

Video of EMG responses to the 0 Hz difference-frequency triplet, using a 500 kHz acoustic field at 2.5 MPa and an independently applied 500 kHz electric field at 20 V. The video shows responses to combined acoustoelectric stimulation, acoustic stimulation alone, and electric field stimulation alone, sequentially.

Supplementary Video 2

Video of EMG responses to the 0.5 Hz difference-frequency triplet, using a 500 kHz acoustic field at 2 MPa and an independently applied 500.005 kHz electric field at 9 V. The video shows responses to combined acoustoelectric stimulation, acoustic stimulation alone, and electric field stimulation alone, sequentially.

Supplementary Video 3

Video of EMG responses to a 500 kHz, 1 MPa acoustic field delivered as a 50% duty-cycle, 0.5 s pulse train. Responses are shown first without the acoustically transparent and electrically attenuating F21 layer in place, followed by the same stimulation with F21 in place to attenuate electric field propagation from the ultrasound transducer.

Supplementary Video 4

Video of EMG responses during the acoustic disconnection test. Responses to a 500 kHz, 1 MPa acoustic field delivered as a 50% duty-cycle, 0.5 s pulse train are shown first with the acoustic path connected and then with the acoustic path disconnected.
