# Supplementary material for: Non-invasive in vivo acoustoelectric neuromodulation and its contribution to ultrasound stimulation
Source: Nat Commun. 2026 Jun 17;17:5073. doi: 10.1038/s41467-026-73826-2 (PMC13276402; doi:10.1038/s41467-026-73826-2)
Supplement: Supplementary file 7 — Reporting Summary [file 41467_2026_73826_MOESM7_ESM.pdf]

Corresponding author(s): Jean Rintoul
Last updated by author(s): Apr 29, 2026

Reporting Summary

Nature Portfolio wishes to improve the reproducibility of the work that we publish. This form provides structure for consistency and transparency in reporting. For further information on Nature Portfolio policies, see our Editorial Policies and the Editorial Policy Checklist.

Statistics

For all statistical analyses, confirm that the following items are present in the figure legend, table legend, main text, or Methods section.

n/a	Confirmed	
☐	☒	The exact sample size (*n*) for each experimental group/condition, given as a discrete number and unit of measurement
☐	☒	A statement on whether measurements were taken from distinct samples or whether the same sample was measured repeatedly
☐	☒	The statistical test(s) used AND whether they are one- or two-sided<br>*Only common tests should be described solely by name; describe more complex techniques in the Methods section.*
☐	☒	A description of all covariates tested
☐	☒	A description of any assumptions or corrections, such as tests of normality and adjustment for multiple comparisons
☐	☒	A full description of the statistical parameters including central tendency (e.g. means) or other basic estimates (e.g. regression coefficient) AND variation (e.g. standard deviation) or associated estimates of uncertainty (e.g. confidence intervals)
☐	☒	For null hypothesis testing, the test statistic (e.g. F, t, r) with confidence intervals, effect sizes, degrees of freedom and P value noted<br>*Give P values as exact values whenever suitable.*
☒	☐	For Bayesian analysis, information on the choice of priors and Markov chain Monte Carlo settings
☒	☐	For hierarchical and complex designs, identification of the appropriate level for tests and full reporting of outcomes
☐	☒	Estimates of effect sizes (e.g. Cohen's *d*, Pearson's *r*), indicating how they were calculated

Our web collection on statistics for biologists contains articles on many of the points above.

Software and code

Policy information about availability of computer code

Data collection	Code supporting the findings in this article is available at (https://github.com/Acoustoelectric/Acoustoelectric_neuromodulation_and_its_contribution_to_US_stimulation), with corresponding datasets at DOI: 10.6084/m9.figshare.c.7909283.
Data analysis	Signal processing and data analysis were performed using custom Python (v3.13.7) scripts that utilised functions from the NumPy (v2.2.6), SciPy (v1.16.3), and Pandas (v2.3.3) Python libraries.

For manuscripts utilizing custom algorithms or software that are central to the research but not yet described in published literature, software must be made available to editors and reviewers. We strongly encourage code deposition in a community repository (e.g. GitHub). See the Nature Portfolio guidelines for submitting code & software for further information.

Data

Policy information about availability of data

All manuscripts must include a data availability statement. This statement should provide the following information, where applicable:

- Accession codes, unique identifiers, or web links for publicly available datasets
- A description of any restrictions on data availability
- For clinical datasets or third party data, please ensure that the statement adheres to our policy

The data supporting the findings of this study are available within the paper, its Supplementary Information and the Source Data file. The raw and analysed datasets

generated during this study have been deposited in Figshare under DOI: 10.6084/m9.figshare.c.7909283.

Research involving human participants, their data, or biological material

Policy information about studies with human participants or human data. See also policy information about sex, gender (identity/presentation), and sexual orientation and race, ethnicity and racism.

Reporting on sex and gender	No humans were used in this study.
Reporting on race, ethnicity, or other socially relevant groupings	No socially relevant groupings were used as no humans were used in this study.
Population characteristics	No humans were used in this study.
Recruitment	No recruiting was required as no humans were used in this study.
Ethics oversight	Human ethics approval was not relevant for this study as it does not involve humans.

Note that full information on the approval of the study protocol must also be provided in the manuscript.

Field-specific reporting

Please select the one below that is the best fit for your research. If you are not sure, read the appropriate sections before making your selection.

- [x] Life sciences
- [ ] Behavioural & social sciences
- [ ] Ecological, evolutionary & environmental sciences

For a reference copy of the document with all sections, see nature.com/documents/nr-reporting-summary-flat.pdf

Life sciences study design

All studies must disclose on these points even when the disclosure is negative.

Sample size	No statistical method was used to predetermine sample size. Sample sizes were determined by the feasibility of the in vivo mouse preparation, the limited lightening-anaesthesia window in which EMG responses could be evoked, and the number of successful counterbalanced trials obtained per animal. Animals were not randomly assigned to separate intervention groups because stimulation conditions were tested within-subject. Stimulation conditions were applied in counterbalanced order where indicated to reduce confounding by anaesthesia-depth drift. Investigators were blinded to the counterbalanced condition order during acquisition and outcome assessment; condition labels were assigned after analysis. Blinding was not possible for hardware setup and stimulation preparation.
Data exclusions	Data were excluded from statistical analyses only when anaesthesia was too deep to evoke an EMG response despite the presence of the Δf frequency in the brain, as described in Supplementary Note S3. Otherwise, files were retained in the dataset, including recordings with poor signal quality or electrochemical offsets, unless a panel-specific criterion is explicitly stated. For the Fig. 4 frequency-specificity analysis, recordings were included when the 1 Hz brain-signal amplitude exceeded the 0.5 Hz amplitude to reduce contamination by electrochemical or pressure-onset effects.
Replication	Findings were replicated across multiple mice and repeated trials as indicated in the figure legends. Phantom measurements were repeated as technical measurements at each frequency, pressure, voltage or spatial position as indicated in the relevant figure legends and Source Data file.
Randomization	Animals were not randomly assigned to separate intervention groups because stimulation conditions were tested within-subject. Stimulation conditions were applied in counterbalanced order where indicated to reduce confounding by anaesthesia-depth drift.
Blinding	Investigators were not blinded to allocation during experiments or outcome assessment.

Reporting for specific materials, systems and methods

We require information from authors about some types of materials, experimental systems and methods used in many studies. Here, indicate whether each material, system or method listed is relevant to your study. If you are not sure if a list item applies to your research, read the appropriate section before selecting a response.

Materials & experimental systems

n/a	Involved in the study
☒	☐ Antibodies
☒	☐ Eukaryotic cell lines
☒	☐ Palaeontology and archaeology
☐	☒ Animals and other organisms
☒	☐ Clinical data
☒	☐ Dual use research of concern
☒	☐ Plants

Methods

n/a	Involved in the study
☒	☐ ChIP-seq
☒	☐ Flow cytometry
☒	☐ MRI-based neuroimaging

Animals and other research organisms

Policy information about studies involving animals; ARRIVE guidelines recommended for reporting animal research, and Sex and Gender in Research

Laboratory animals	Wild-type C57BL/6J mice, male and female, aged 3–6 months, obtained from Charles River Laboratories, were used. Mice were housed in standard cages in the Imperial College London animal facility with ad libitum access to food and water in a controlled light-dark cycle environment and were monitored by veterinary staff.
Wild animals	The study did not involve wild animals.
Reporting on sex	Both male and female mice were included. Sex was not used as a biological variable in the statistical analysis because the study was designed to test the physical requirement for simultaneous acoustic and electric fields rather than sex-specific differences, and sample sizes were not powered for sex-disaggregated comparisons.
Field-collected samples	The study did not involve samples collected from the field.
Ethics oversight	All animal procedures were approved by the Imperial College London Animal Welfare and Ethical Review Body under project licence PPL P2EA80855 and were performed in accordance with the United Kingdom Animals (Scientific Procedures) Act 1986.

Note that full information on the approval of the study protocol must also be provided in the manuscript.

Plants

Seed stocks	The study did not involve seed stocks.
Novel plant genotypes	The study did not involve plant genotypes
Authentication	The study did not involve plants so authentication of seed stocks was not required.
