# Supplementary material for: Non-invasive in vivo acoustoelectric neuromodulation and its contribution to ultrasound stimulation
Source: Nat Commun. 2026 Jun 17;17:5073. doi: 10.1038/s41467-026-73826-2 (PMC13276402; doi:10.1038/s41467-026-73826-2)
Supplement: Supplementary file 8 — Transparent Peer Review file [file 41467_2026_73826_MOESM8_ESM.pdf]

nature portfolio

Peer Review File

Non-invasive in vivo acoustoelectric neuromodulation and its contribution to ultrasound stimulation

Corresponding Author: Dr Jean Rintoul

This file contains all reviewer reports in order by version, followed by all author rebuttals in order by version.

Version 0:

Reviewer comments:

Reviewer #1

(Remarks to the Author)
This study demonstrated a non-invasive neuromodulation technique that combines ultrasonic and electrical modalities to achieve effective, focal neural stimulation. The authors explored acoustoelectric signals, which depend on the difference between acoustic and electrical frequencies, using both phantom and in vivo setups. The study successfully demonstrated motor-evoked potential with unilateral forelimb movement, showing stronger, more robust responses to acoustoelectric stimulation than to pressure-only (acoustic) or voltage-only (electrical) stimulation. In addition, this work offers a new perspective on the mechanism of ultrasound neuromodulation by investigating the acoustoelectric interaction between ultrasound and its electric artefact and its contribution to ultrasound neuromodulation, which may help better elucidate the mechanism of ultrasound stimulation. However, there are still some shortcoming that need to be addressed prior to publication.

Comments
Abstract: The abstract appears to be very general. Can the authors add some quantitative metrics of modulation as well as the acoustoelectric parameter range that they studied?
Line 91, in Fig 2d, please add in vitro prior to “Setup”
Line 143: Methods should describe the protocol for the lightning anesthesia window.
In Fig 3 and video supplement 1, forelimb movement in response to both acoustoelectric and acoustic-only stimulation appears unilateral. Was the target engagement in the motor cortex with the acoustic field also unilateral?
Between Fig 3 and 4, any reasons not to keep the acoustic pressure the same?
Voltage-only is at 500 kHz in Fig 3 and at 500.001kHz in Fig 4, and EMG responses are completely different; no response at 500 kHz (even with a higher voltage of 20V) but significant at 500.001 kHz (12V). Why?
The evoked responses to pressure-only between fig 3 and fig 4 are very different. You may want to use the same pressure as the pressure used in fig 4 is 1.8 MPa, and the pressure used in fig 3 is 2.5 MPa. Overall, it is difficult to compare DC (Δf=0Hz) and AC (Δf=1Hz) neuromodulation because of differences in the amplitudes of pressure and voltage.
In Fig 4d demonstrated 3 time-correlated peaks are demonstrated, which are interesting observations. However, zero latency between brain and EMG responses doesn’t make a lot of sense, as they have a causal relationship (i.e., neural activity in the motor cortex evoked by acoustoelectric stimulation results in muscle contraction in forelimbs).
Please address the discrepancy between Fig 4 (Δf=1Hz) and video supplement 2 (Δf=0.5Hz)
In Fig 3 and 4, please use consistent terminology, either acoustic-only or pressure-only.
Line 337, how much is the amplitude of the electric field attenuated by F21? And how does it correlate with the evoked responses?
In Fig 6, are Vus signals attenuated by F21? Then, the decreases in the evoked responses may be due to ultrasound stimulation at lower pressure, resulting from the attenuated RF input?
In Figures 6 and 7, can you quantify the electric artefact from the transducer? How does it compare to the electric field you applied to in the experiment in Fig 3 (20V) and Fig 4 (12V)?

(Remarks on code availability)

Reviewer #2

(Remarks to the Author)
Authors describe a new mechanism for neural stimulation in the rat brain based on the acoustoelectric effect that combines focused ultrasound with a high frequency externally applied electromagnetic field. The multiplication of the two energy fields produces sum and difference frequencies with the latter contributing to neuronal modulation of the motor cortex. The approach has the potential advantage of improving the selectivity of noninvasive brain stimulation compared to tFUS or tDCS/tACS alone by introducing two separate, controllable external vector fields for stimulation (pressure and RF). The authors perform meticulous control experiments to document and account for potential artifacts present in their methodology. The authors further postulate that the acoustoelectric effect may play a role in tFUS neural stimulation since a near-DC potential/current is induced due to the heterodyning of the US frequency with the low frequency physiologic currents. The paper is well written, and the figures are clear and easy to follow. Answers to the following questions will improve the quality of the manuscript and help understand AES.

1) Based on theory, what is the estimated amplitude of the induced acoustoelectric currents at the US focus? How does this compare to electrical stimulation, tACS/tDCS or TMS?
2) What is the source of the strong DC component in Fig 2i,ii? Why does Vae seem to reach a maximum after ~3 seconds (similar to the temperature plot in Fig. 5b)?
3) Results would be more convincing and quantitative (activation threshold etc,) if the amplitudes of the focal pressure and external fields were systematically varied. Do the authors have any additional information that describes the activation threshold (or activation curve) for tFUS (US alone) and AES on the evoked brain and EMG responses? Figures 3 and 4 suggest AES has less variability than tFUS, although tFUS alone is able to generate a similar maximum amplitude of activation.
4) The authors report a temperature increase from the US exposure, stating this exceeded safety parameters. Can authors please report the exposure in terms of standard safety measures based on the US intensity (e.g, Isppa, Ispta, Isata etc)?
5) Why does the amplitude of VAE depend on the difference frequency? Is this physical or physiologic in origin? Can it be predicted based on theory?
6) What is the spatial dependency (location of US focus in brain) on evoked responses? Was the US focal spot moved one beam width away from motor cortex to determine whether the stimulation effect was reduced/eliminated?
7) Please include something quantitative in the abstract. What are the take-home numbers, such as the threshold of AES or AES decreased the tFUS threshold by XX (e.g., US and E-field amplitudes, what is the spatial selectivity in mm)?
8) What is the clinical potential of AES? What are the barriers?
9) The peer-reviewed paper connected to reference #16 (Alvarez et al. 2020) is Alvarez et al. "In vivo acoustoelectric imaging for high-resolution visualization of cardiac electric spatiotemporal dynamics." Appl Opt. 2020 Dec 20;59(36):11292-11300. doi: 10.1364/AO.410172.

(Remarks on code availability)
I verified the code is present and documented. I did not otherwise evaluate the code or files.

Reviewer #3

(Remarks to the Author)
In this manuscript, Rintoul et al. demonstrate a non-invasive neuromodulation approach that combines electrical and mechanical (ultrasound) waves to achieve electrically focused stimulation. The authors propose that small, local ion conductivity changes driven by mechanical waves can induce voltage gradients in the presence of an applied electric field. Furthermore, this multimodal interaction can generate signals with frequencies equal to the difference between the principal waves, which may serve as a mechanism for neuromodulation.
Overall, this concept is quite interesting from both physical and engineering perspectives. However, there are major concerns regarding the significance and accuracy of the results presented.
The primary motivation for establishing such a multimodal system is to achieve direct electrical stimulation with a spatial resolution comparable to focused ultrasound. In no part of the manuscript do the authors provide information regarding the in vivo spatiotemporal resolution of the stimulation. While the videos are informative, it is evident that the effects of stimulation are highly widespread, including bilateral motor twitches.
Given the novelty of the experimental setup, clearer schematics and real experimental photographs would greatly improve the accessibility of the manuscript. The current schematic is confusing; the authors may consider developing a schematic centered on a coronal slice of the brain and skull.
In Figure 1b, the authors apply approximately 20 V (according to the scale bar) at f = 500 kHz, which results in no measurable changes in either LFP or EMG (E only). In contrast, in Figure 4a, a smaller voltage amplitude (6 V) applied at f = 500.001 kHz results in a measurable EMG response. Furthermore, similar stimulations (500Khz US) have very different EMG profile. Authors should carefully address these inconsistencies.
Despite the inclusion of multiple control experiments, the current results do not conclusively demonstrate effective local stimulation. Given the novelty of this approach, additional neural spiking analyses—such as post-stimulus time histograms of unit firing and coherence between firing rates and stimulation frequencies—are necessary to support the claims.
What is the magnitude of the ion concentration changes induced by the mechanical waves? Based on the data presented for acoustoelectric stimulation, the resulting electric field appears to be on the order of hundreds of microvolts, which is significantly lower than what is typically required for neural entrainment.
Overall, this is a very intriguing finding; however, the current data do not adequately support the stated claims, and multiple additional experiments are required to substantiate the conclusions.

(Remarks on code availability)

Version 1:

Reviewer comments:

Reviewer #1

(Remarks to the Author)

The authors did an excellent job responding to the reviews.

(Remarks on code availability)

Reviewer #2

(Remarks to the Author)
The authors have thoroughly responded to criticisms and made appropriate edits to the manuscript. I have no further comments or questions.

(Remarks on code availability)

Reviewer #3

(Remarks to the Author)
Authors were able to address all of my comments, and I have no further questions.

(Remarks on code availability)

Open Access This Peer Review File is licensed under a Creative Commons Attribution 4.0 International License, which permits use, sharing, adaptation, distribution and reproduction in any medium or format, as long as you give appropriate credit to the original author(s) and the source, provide a link to the Creative Commons license, and indicate if changes were made.
In cases where reviewers are anonymous, credit should be given to 'Anonymous Referee' and the source.
The images or other third party material in this Peer Review File are included in the article’s Creative Commons license, unless indicated otherwise in a credit line to the material. If material is not included in the article’s Creative Commons license and your intended use is not permitted by statutory regulation or exceeds the permitted use, you will need to obtain

permission directly from the copyright holder.
To view a copy of this license, visit https://creativecommons.org/licenses/by/4.0/

Dear Reviewers,

We thank you for your careful, constructive, and insightful evaluation of our manuscript. We are grateful for the time and effort you devoted to reviewing our work and for the helpful suggestions provided.

Below, we respond point by point to each comment. Reviewer comments are shown in black, and our responses are shown in blue.

Sincerely,

Jean Rintoul, PhD

Reviewer #1:

This study demonstrated a non-invasive neuromodulation technique that combines ultrasonic and electrical modalities to achieve effective, focal neural stimulation. The authors explored acoustoelectric signals, which depend on the difference between acoustic and electrical frequencies, using both phantom and in vivo setups. The study successfully demonstrated motor-evoked potential with unilateral forelimb movement, showing stronger, more robust responses to acoustoelectric stimulation than to pressure-only (acoustic) or voltage-only (electrical) stimulation. In addition, this work offers a new perspective on the mechanism of ultrasound neuromodulation by investigating the acoustoelectric interaction between ultrasound and its electric artefact and its contribution to ultrasound neuromodulation, which may help better elucidate the mechanism of ultrasound stimulation. However, there are still some shortcomings that need to be addressed prior to publication.

Comments

Reviewer 1, Comment 1: Abstract: The abstract appears to be very general. Can the authors add some quantitative metrics of modulation as well as the acoustoelectric parameter range that they studied?

Response: We thank the reviewer for this suggestion. We have revised the abstract to include explicit quantitative metrics and parameter ranges characterising the acoustoelectric stimulation regime studied. Specifically, the abstract now reports the acoustic frequency and pressure used (500 kHz, 1 MPa), the measured in vivo acoustoelectric potential amplitude ≈9 mV, the corresponding estimated focal electric field ~6 V/m, and the millimetre-scale spatial confinement demonstrated in controlled phantom experiments. Because response amplitude and probability were strongly state-dependent under anaesthesia, we did not report a single activation threshold; instead, we provide field magnitudes, frequencies, and spatial scales that directly define the operating regime of acoustoelectric neuromodulation. The revised abstract text is provided below:

‘Non-invasive brain stimulation offers therapeutic potential without surgery, yet existing electrical approaches lack spatial precision due to the long wavelengths of electric fields. Here we demonstrate acoustoelectric neuromodulation, a nonlinear interaction between applied acoustic and electric fields that generates spatially localized, low-frequency electric fields at the ultrasound

focus. Using in vitro and in vivo rodent electrophysiology, we show motor-evoked responses that depend on both the amplitude and frequency of the acoustoelectric field, with controls excluding purely acoustic or electrical origins. In vivo measurements show acoustoelectric potentials of ≈9 mV, corresponding to estimated focal electric fields of ~6 V/m at 500 kHz and 1 MPa acoustic pressure, with ~1.5 mm extrema spacing demonstrated in phantom experiments. Importantly, we identify an acoustoelectric contribution to conventional ultrasound stimulation, arising from interactions between ultrasound-induced electrical signals and propagating acoustic waves, establishing acoustoelectric neuromodulation as a distinct mechanism influencing ultrasound-based brain stimulation.'

Reviewer 1, Comment 2: Line 91, in Fig 2d, please add in vitro prior to "Setup"

Response: Revised as requested.

Reviewer 1, Comment 3: Line 143: Methods should describe the protocol for the lightning anaesthesia window.

Response: We agree that this was insufficiently described and have revised the Methods to clarify the experimental protocol during the lightening anaesthesia window. The following text has been added to the Experimental procedure section:

"Experimental procedure. Anaesthesia was induced via subcutaneous injection of ketamine (75 mg/kg) and xylazine (10 mg/kg). EMG responses could only be elicited during the period in which anaesthesia depth lightened, typically occurring approximately 45–60 minutes after administration. Prior to this window, no EMG activity was observed regardless of stimulation amplitude. Beginning approximately 30 minutes after anaesthesia induction, a 1 MPa acoustic stimulus was applied at 5-minute intervals to monitor the transition into the lightening anaesthesia window. As soon as EMG movements were elicited, the experiments commenced. Once small spontaneous movements were observed, the experiment was terminated."

Reviewer 1, Comment 4: In Fig 3 and video supplement 1, forelimb movement in response to both acoustoelectric and acoustic-only stimulation appears unilateral. Was the target engagement in the motor cortex with the acoustic field also unilateral?

Response: We cannot infer unilateral cortical target engagement from unilateral forelimb movement under these conditions. At 500 kHz, the acoustic wavelength in brain tissue (~3 mm) is a substantial fraction of mouse head dimensions, and skull/air boundary reflections can produce standing-wave patterns and multiple intracranial pressure maxima, reducing spatial confinement (**Supplementary Note 4**). We have clarified this limitation in the revised manuscript and support it with finite-element simulations in a realistic mouse head model (Sim4Life), which show a clear focus under acoustically matched boundary conditions but complex multi-maxima patterns when boundary reflections are present (**Fig. S4**).

This clarification has been added to the Results section describing Fig. 3, immediately prior to the concluding sentence of that section, with supporting simulations referenced in Supplementary Note 4.

'Although EMG responses in **Fig. 3** and **Video Supplement 1** appear unilateral, unilateral forelimb movement cannot be taken as evidence of unilateral cortical target engagement under

these conditions. At the ultrasound carrier frequency used here (500 kHz), the acoustic wavelength in brain tissue (~3 mm) is a substantial fraction of mouse head dimensions, and skull–air boundary reflections can generate standing waves and multiple intracranial pressure maxima. Finite-element simulations in a realistic mouse head model (**Supplementary Note 4**) show that, while a clear focal region is present under acoustically matched boundary conditions, complex multi-maxima pressure distributions arise when boundary reflections are present, limiting spatial confinement in vivo'.

Reviewer 1, Comment 5: Between Fig 3 and 4, any reasons not to keep the acoustic pressure the same? Voltage-only is at 500 kHz in Fig 3 and at 500.001kHz in Fig 4, and EMG responses are completely different; no response at 500 kHz (even with a higher voltage of 20V) but significant at 500.001 kHz (12V). Why? The evoked responses to pressure-only between fig 3 and fig 4 are very different. You may want to use the same pressure as the pressure used in fig 4 is 1.8 MPa, and the pressure used in fig 3 is 2.5 MPa. Overall, it is difficult to compare DC (Δf=0Hz) and AC (Δf=1Hz) neuromodulation because of differences in the amplitudes of pressure and voltage.

Response: We thank the reviewer for this careful observation. The stimulus parameters in **Fig. 3** and **Fig. 4** were intentionally different because the two figures address distinct mechanistic questions rather than providing a direct quantitative comparison between Δf = 0 Hz and Δf = 1 Hz conditions.

Mechanistic aim: Fig. 3 tests whether an independently applied high-frequency electric field can modulate stimulation when mixed with ultrasound at the same carrier frequency (Δf = 0 Hz). **Fig. 4** tests whether the frequency content of the acoustoelectrically generated field can be independently controlled by offsetting the electric-field carrier (500.001 kHz) to produce a 1 Hz difference-frequency component (Δf = 1 Hz). We have revised the figure legends/text to make this distinction explicit and to avoid implying a direct amplitude comparison between **Fig. 3** and **Fig. 4**.

Why lower pressure/voltage in Fig. 4: We empirically found that the 1 Hz modulation was most clearly expressed at lower acoustic pressures (and correspondingly reduced applied drive voltage) because higher-pressure conditions introduced a large DC onset/offset component that dominated the low-frequency response. As discussed in the revised manuscript, this arose from a competing Δf = 0 Hz contribution (including parasitic/capacitive coupling-related DC offsets), which produced strong transient responses that obscured the steady-state 1 Hz periodic modulation. Accordingly, **Fig. 4** prioritizes frequency specificity (Δf = 1 Hz) rather than providing a direct amplitude comparison with the Δf = 0 Hz conditions in **Fig. 3**.

Clarifying the "voltage-only" and "pressure-only" traces: The reviewer is correct that single-trial EMG traces can appear different across figures. EMG responses varied across time due to fluctuating physiological conditions (including anaesthesia depth), which is well documented in the focused ultrasound literature[1]. Therefore, the single traces shown in **Fig. 3** and **Fig. 4** are intended as illustrative examples rather than universally "representative" waveforms across the entire dataset. To avoid confusion, we have revised the text to use "example/illustrative trace" and to emphasize that the primary comparisons are made within counterbalanced triplets acquired under matched physiological conditions (voltage-only, pressure-only, and combined trials at the same acoustic pressures and voltage outputs), rather than across figures or across time-separated experiments.

Consistency of conclusions: Importantly, the key conclusion is not that voltage-only or pressure-only never evokes a response, but that these conditions did not evoke consistent,

reliable EMG modulation matching the targeted difference-frequency component, whereas the combined (E+US) condition produces an acoustoelectrically generated low-frequency field that tracks the measured in-brain signal (V_{BRAIN}) and is accompanied by corresponding EMG modulation. We have revised the wording in the Results/legend to replace absolute statements (e.g., “no response”) with “not consistently evoked / not reliably present at the group level,” where appropriate.

Text added (**Fig. 4**): “We found that the 1 Hz difference frequency was most clearly expressed in the EMG when ultrasound pressure was reduced to minimize large DC onset and offset components, which otherwise dominated the low-frequency response. Accordingly, **Fig. 4** is designed to test frequency specificity of acoustoelectric stimulation (Δf = 1 Hz), rather than to provide a direct amplitude comparison with DC (Δf = 0 Hz) conditions shown in **Fig. 3**.”

Reviewer 1, Comment 6: In Fig 4d demonstrated 3 time-correlated peaks are demonstrated, which are interesting observations. However, zero latency between brain and EMG responses doesn’t make a lot of sense, as they have a causal relationship (i.e., neural activity in the motor cortex evoked by acoustoelectric stimulation results in muscle contraction in forelimbs). Please address the discrepancy between Fig 4 (Δf=1Hz) and video supplement 2 (Δf=0.5Hz)

Response: We thank the reviewer for raising this important point. We agree that a strict interpretation of zero latency between cortical activity and EMG would be physiologically implausible. Importantly, **Fig. 4 d** does not imply zero-latency causation: in our peak-alignment analysis, peaks were considered “aligned” if they occurred within 0.2 s (1/5 of the 1 Hz period), which is orders of magnitude larger than expected corticospinal conduction delays (ms). Thus, the figure indicates coarse temporal coincidence within the stimulation cycle, not instantaneous propagation from cortex to muscle.

Rather, the EMG responses observed here are threshold- and gradient-dependent events, not responses tied to the midpoint or phase of the acoustoelectrically generated sinusoid. In **Fig. 4 d**, EMG activity is evoked preferentially during periods in which the acoustoelectric waveform exhibits the largest rate of change, corresponding to a larger induced electric field over a shorter time interval. Subsequent lower-amplitude or more slowly varying portions of the sinusoid do not reliably evoke EMG responses, resulting in discrete, time-correlated peaks rather than continuous phase-locked activity.

A similar pattern is observed in **Video Supplement 2** (Δf = 0.5 Hz), where EMG responses are strongest during intervals of maximal gradient change rather than at a fixed phase of the sinusoidal envelope. Thus, the apparent discrepancy between Δf = 1 Hz and Δf = 0.5 Hz reflects differences in the temporal spacing of threshold-crossing events, not differences in the underlying mechanism.

This interpretation is consistent with established electrical stimulation literature, in which neural and muscular activation is governed by the spatial and temporal gradients of the induced electric field (V/m), rather than by field amplitude alone, as described by classical activating-function and cable-theory models[2]. In addition, repeated stimulation is known to produce EMG habituation[3], leading to a progressive reduction in response amplitude with repeated or sustained activation, which further contributes to the observed pattern of discrete responses over time.

We have clarified these points in the revised manuscript to avoid overinterpretation of apparent zero-latency alignment and to more accurately reflect the physiological constraints on neural-to-muscle transmission.

Added into the text for **Fig. 4**:
'At Δf = 1 Hz, EMG responses were temporally sparse and non-sinusoidal, reflecting threshold-based activation rather than phase-locked entrainment, with physiological factors such as EMG habituation contributing to the observed response pattern. DC components were occasionally observed, likely arising from a combination of standing acoustic waves in the mouse head (**Supplementary Note 4**), electrochemical offsets at the electrode interface (**Supplementary Note 5**), and onset responses to the acoustic field alone (**Fig. 3b**, pressure-only condition).'

Reviewer 1, Comment 7: In Fig 3 and 4, please use consistent terminology, either acoustic-only or pressure-only.

Response: We agree and have standardized terminology throughout the manuscript, figure panels, and captions to use "pressure-only" (for ultrasound-only) and "voltage-only" (for electric-only), with "acoustoelectric" for combined stimulation. All instances of "acoustic-only" have been replaced accordingly (Results and Figs. 3–4 legends/captions).

Reviewer 1, Comment 8: Line 337, how much is the amplitude of the electric field attenuated by F21? And how does it correlate with the evoked responses?

Response: We agree that this point was insufficiently explained and have revised line 337 accordingly. Calibration measurements performed with and without F21 in place (**Supplementary Note 2**) show that placing a 2-mm-thick F21 layer between the end of the transducer cone and the acoustic coupling gel results in a 90% attenuation of the transducer-induced electric field amplitude in an *in vitro* phantom. Consistent attenuation is also observed in vivo, as shown in **Supplementary Fig. S8a–c**, which directly quantifies the reduction in the electric field measured at the recording electrodes when F21 is present.

Updated in Results text for **Fig. 6**:
'During the lightening anaesthesia window 2mm thick F21 was inserted into the gel below the ultrasound cone to shield the electrical artefact coming from the ultrasound transducer, decreasing the amplitude of the electric field present in the medium by 90% (**Supplementary Note 2 b**).'

To address correlation with evoked responses, we performed paired within animal comparisons (F21 vs no-F21 at matched acoustic pressure). In these paired trials, reducing the carrier artefact amplitude with F21 is accompanied by a reduction in both the low-frequency brain response (V_{BRAIN}) and EMG amplitude (**Fig. 6d(ii–iii)**). By contrast, electrical coupling in the absence of an acoustic wave (air-gap control) does not evoke EMG despite a measurable carrier artefact (**Fig. 7**), indicating that the carrier amplitude alone is insufficient. Together, these results support that the evoked responses track the co-presence of acoustic and electric fields (consistent with an acoustoelectric contribution) rather than the electrical artefact magnitude in isolation.

Updated in Results text for **Fig. 6**:
'Across paired trials, attenuation of the carrier artefact with F21 was accompanied by a reduction in both the low-frequency brain response (V_{BRAIN}) and the evoked EMG amplitude, consistent with an acoustoelectric contribution under these stimulation conditions.'

Reviewer 1, Comment 9: In Fig 6, are V_{US} signals attenuated by F21? Then, the decreases in the evoked responses may be due to ultrasound stimulation at lower pressure, resulting from the attenuated RF input?

V_{US} (the voltage applied to the ultrasound transducer) is not attenuated by F21. As shown in **Fig. 6b** (upper panel), the monitored transducer drive V_{US} is maintained and was in fact increased by ~15% when F21 was inserted, to compensate for the small, reproducible attenuation of acoustic pressure introduced by the 2 mm F21 layer (**Supplementary Note 2b**). This ensured that the delivered acoustic pressure at the coupling interface/medium was matched between F21 and no-F21 conditions.

What F21 does attenuate is the transducer-drive electrical artefact measured at the intracranial electrodes (quantified in **Fig. 6d(i)** and **Fig. 7c** and **Supplementary Fig. S8**), while keeping acoustic pressure matched. Therefore, the reduction in evoked responses observed with F21 cannot be explained by reduced ultrasound pressure due to lower transducer drive and is instead associated with attenuation of the intracranially measured electrical artefact under pressure-matched conditions.

We have clarified these points in the revised manuscript to distinguish (i) the monitored transducer drive V_{US} from (ii) the artefact measured at the intracranial electrodes.

This results text for **Fig. 6** has been updated:
'During the lightening anaesthesia window, a 2 mm F21 layer was inserted into the gel below the ultrasound cone to reduce the transducer-drive electrical artefact recorded at the intracranial electrodes. The insertion of the 2 mm F21 layer produced a small, reproducible attenuation of acoustic pressure (**Supplementary Note 2b**); therefore, in all experiments the transducer drive V_{US} was increased by 15% when F21 was present (**Fig. 6b**), ensuring that delivered acoustic pressure in the brain was matched across F21 and no-F21 conditions. The F21 layer was then removed and the procedure repeated 1–2 times per mouse, alternating trial order to minimise anaesthesia depth confounds (**Supplementary Note 3**).'

Reviewer 1, Comment 10: In Figures 6 and 7, can you quantify the electric artefact from the transducer? How does it compare to the electric field you applied to in the experiment in Fig 3 (20V) and Fig 4 (12V)?

Response: Yes. We quantify the transducer-drive electrical artefact as the carrier-frequency component measured at the intracranial electrodes. Across the pulsed tFUS experiments, this artefact is on the order of a few millivolts at the recording electrodes (**Fig. 6d i**: 4601 ± 2116 μV without F21; 2895 ± 1253 μV with F21; n = 36 pulses across 7 mice), with a 7 mm inter-electrode spacing, these correspond to electric-field estimates of 0.66 V/m (no F21) and 0.41 V/m (with F21), respectively ($E \approx \frac{V}{d}$). In the acoustic disconnection (air-gap) control, the carrier artefact remains measurable but is smaller (**Fig. 7c**: 3066 ± 2302 μV gap vs 5967 ± 4773 μV no-gap; n = 36), corresponding to 0.44 V/m (gap) and 0.85 V/m (no-gap) across the same 7 mm spacing.

The independently applied stimulation voltages in **Fig. 3** (20 Vpp) and **Fig. 4** (12 Vpp) refer to the generator/amplifier outputs driving the external stimulation electrodes, not to the intracranial electrode-recorded amplitude at the applied carrier frequency. Because coupling depends on geometry and impedance, the relationship between drive voltage and intracranial carrier amplitude is not 1:1. In addition, during the independent-field experiments we used a front-end hardware filter that attenuates the carrier by −150 dB at 500 kHz (**Supplementary Note 12**), i.e. by a factor of $10^{\frac{-150}{20}} \approx 3.16 \times 10^{-8}$. Therefore, any carrier component measured at the digitizer

in those experiments corresponds to an estimated intracranial carrier that is larger by $10^{\frac{150}{20}} \approx 3.16 \times 10^7$ before filtering, and the corresponding electric field can be estimated as $E \approx (V_{meas} \times 10^{\frac{150}{20}})/7mm$. We have clarified these distinctions in the revised text.

Reviewer #2:

Authors describe a new mechanism for neural stimulation in the rat brain based on the acoustoelectric effect that combines focused ultrasound with a high frequency externally applied electromagnetic field. The multiplication of the two energy fields produces sum and difference frequencies with the latter contributing to neuronal modulation of the motor cortex. The approach has the potential advantage of improving the selectivity of non-invasive brain stimulation compared to tFUS or tDCS/tACS alone by introducing two separate, controllable external vector fields for stimulation (pressure and RF). The authors perform meticulous control experiments to document and account for potential artifacts present in their methodology. The authors further postulate that the acoustoelectric effect may play a role in tFUS neural stimulation since a near-DC potential/current is induced due to the heterodyning of the US frequency with the low frequency physiologic currents. The paper is well written, and the figures are clear and easy to follow. Answers to the following questions will improve the quality of the manuscript and help understand AES.

Comments

Reviewer 2, Comment 1: Based on theory, what is the estimated amplitude of the induced acoustoelectric currents at the US focus? How does this compare to electrical stimulation, tACS/tDCS or TMS?

Response: Based on theory, the amplitude of the induced acoustoelectric field can be estimated from the governing relation

$$\nabla \cdot \vec{E}_{ae} = -k\overrightarrow{\nabla P} \cdot \vec{E}_o$$

where $\overrightarrow{\nabla P}$ is the pressure gradient, $\vec{E}_o$ is the externally applied electric field, and $\vec{E}_{ae}$ is the acoustoelectrically induced modulation of the electric field. The proportionality constant k captures pressure-dependent changes in ionic mobility and compressibility; a full derivation is provided in the Supplementary Material of our prior work in Communications Physics[4]. Consistent with earlier experimental and theoretical studies by Lavandier and Jossinet[5], we found that the ionic mobility contribution dominates the acoustoelectric response in conductive biological media, indicating the conductivity distribution within the tissue will impact the acoustoelectric amplitude. Using the *in vivo* mouse brain acoustoelectric measurements shown in **Fig. 3** to estimate neuromodulation amplitude feasibility, we observe an acoustoelectric peak-to-peak onset transient of 9.1 mV (**Fig 3. b**). From spatial acoustoelectric maps (**Fig 2. b,c**), the distance between the extrema corresponds to approximately half the acoustic wavelength (≈ 1.5 mm at 500 kHz), giving an order-of-magnitude field estimate $E_{AE} = \frac{\Delta V}{\Delta x} \approx 6\, V/m$ depending on the specific condition (and electrode geometry). We have clarified in the manuscript that this estimate is geometry dependent.

To convert this to an induced current density, we use the relation $J_{AE} = \sigma E_{AE}$. Taking brain conductivity in the ~0.2–0.6 S/m range (frequency and tissue-dependent), a focal field in the ~1–6V/m range corresponds to an acoustoelectrically induced current density of

$$J_{AE} \sim \sigma E_{AE} \approx (0.2 - 0.6) \times 6\, A/m^2 \approx 1.2 - 3.6\, A/m^2,$$

as an order-of-magnitude estimate.

Comparison to other modalities: This estimated field magnitude (~6 V/m) is of the same order of magnitude as, and somewhat higher than effective cortical fields reported to modulate excitability in tACS (typically ~0.2–1 V/m in cortex[6]), while remaining below the macroscopic fields associated with TMS (~80–100 V/m at motor threshold in humans[7]). However, direct numerical comparison with TMS should be interpreted cautiously, as the underlying mechanisms and spatial scales differ substantially. TMS relies on electromagnetic induction to generate broadly distributed cortical fields shaped by skull conductivity, cerebrospinal fluid, cortical folding, and axonal orientation[8]. In contrast, acoustoelectric stimulation generates a spatially confined, low-frequency electric field via heterodyning of an independently applied electric field with a focused ultrasound field, such that the low-frequency component is generated predominantly at the pressure focus where ∇P is largest. This process produces localized electric-field gradients that interact with axonal geometries over millimetre length scales, rather than reproducing TMS-like bulk depolarizing fields.

Reviewer 2, Comment 2: What is the source of the strong DC component in Fig 2i,ii? Why does Vae seem to reach a maximum after ~3 seconds (similar to the temperature plot in Fig. 5b)?

Response: The DC component in **Fig. 2f i,ii** arises from difference-frequency mixing at Δf = 0 when an electric field component is present during ultrasound exposure. In our setup, two electric field contributions were present at the focus: (i) the independently applied electric field used for AES, and (ii) an artifactual electric field produced by electrical coupling between the ultrasound transducer/drive electronics and the conductive medium. Both field components can participate in acoustoelectric mixing with the acoustic field and therefore contribute to a DC term. Consistent with this interpretation, the magnitude of the DC component is reduced when the artifactual coupling is eliminated or mitigated (see **Supplementary Note 9 and Fig. 6** for respective in vitro and in vivo demonstrations).

We also observed that the rise of V_{AE} following ultrasound onset is not instantaneous in any of our experiments, with a gradual increase over approximately 1 s before reaching a plateau (**Fig. 2 f i,ii**). Importantly, the dedicated heating control experiments in **Fig. 5a–c** demonstrate that temperature-related effects alone do not reproduce acoustoelectric signal features observed in **Fig. 2**, allowing us to distinguish the observed V_{AE} dynamics from purely thermal drift.

Reviewer 2, Comment 3: Results would be more convincing and quantitative (activation threshold etc,) if the amplitudes of the focal pressure and external fields were systematically varied. Do the authors have any additional information that describes the activation threshold (or activation curve) for tFUS (US alone) and AES on the evoked brain and EMG responses? Figures 3 and 4 suggest AES has less variability than tFUS, although tFUS alone is able to generate a similar maximum amplitude of activation.

Response: We thank the reviewer for raising this important point. Systematic determination of activation thresholds or activation curves in anaesthetized mice is challenging due to pronounced state-dependent variability. In our experiments, response amplitudes were strongly modulated by anaesthesia depth, with identical acoustic stimuli producing markedly different evoked responses within the same animal as anaesthesia lightened. This effect is illustrated in **Supplementary Note 3** and has been reported previously by others[9].

To mitigate this confound, we did not perform extended monotonic sweeps of acoustic pressure or electric field amplitude, as such protocols would be dominated by time-dependent changes in cortical excitability rather than stimulus amplitude. Instead, each experiment was performed using counterbalanced, interleaved stimulus triplets, in which acoustoelectric stimulation (AES), ultrasound-only stimulation (tFUS), and electric-field–only conditions were applied with identical amplitudes. This design allowed relative comparison between stimulation modalities while statistically cancelling time-dependent changes associated with lightening anaesthesia.

Within this framework, both AES and ultrasound-only stimulation could evoke robust EMG responses at matched acoustic pressures (**Figs. 3** and **4**). Rather than defining a single activation threshold, we therefore focus on relative response occurrence and evoked amplitude under controlled, interleaved conditions. This approach enables comparison between stimulation modalities while avoiding over-interpretation of variability that is dominated by anaesthetic state rather than stimulus parameters.

We note that defining a single activation threshold in terms of applied pressure or voltage alone may be insufficient, as stimulation efficacy depends also on the spatial distribution of the induced electric field over relevant neural length scales and orientations. Consistent with classical cable-theory and activating-function models of neural stimulation, activation is determined by spatial derivatives of the extracellular potential along neural elements rather than by scalar field magnitude alone[2]. We have clarified this point in the revised manuscript.

Fig. 4 text first paragraph has been updated to incorporate an explanation of why an amplitude sweep was not performed:

'We next tested neural responses to acoustic and electric fields applied at a difference frequency Δf = 1 Hz (f_{US} = 500 kHz, f_E = 500.001 kHz; n = 9 mice, 4-5 trials per mouse, 44 triplet trials total). Because EMG responses were only observable during a limited lightening-anaesthesia window, trials occurring prior to motor responsiveness were excluded, and variability in trial number reflects physiological state rather than experimental selection. During acoustoelectric stimulation, the 1 Hz difference frequency was present in both the brain-recorded signal and the envelope EMG but was absent when acoustic or electric fields were applied alone (**Fig. 4 a**). We found that the 1 Hz component was most clearly expressed in the EMG when ultrasound pressure was reduced to minimize large DC onset and offset components, which otherwise dominated the low-frequency response. Accordingly, **Fig. 4** is designed to test the frequency specificity of acoustoelectric stimulation (Δf = 1 Hz), rather than to provide a direct amplitude comparison with DC (Δf = 0 Hz) conditions shown in **Fig. 3**. Because evoked responses varied strongly with anaesthesia depth, activation thresholds were not estimated from monotonic amplitude sweeps; instead, stimulation modalities were compared using counterbalanced, interleaved trials at matched amplitudes to assess relative response occurrence and amplitude.'

Reviewer 2, Comment 4: The authors report a temperature increase from the US exposure, stating this exceeded safety parameters. Can authors please report the exposure in terms of standard safety measures based on the US intensity (e.g, Isppa, Ispta, Isata etc)?

Response: We thank the reviewer for requesting standard exposure metrics. The ultrasound was delivered continuously (CW) at 500 kHz with a spatial-peak pressure amplitude of 1 MPa at the focus. For a sinusoidal wave, the spatial-peak intensity is given by $I = \frac{p_{pk}^2}{2\rho c}$. Using $\rho = 1000 kg/m^3$ and $c = 1540 m/s$, this corresponds to a spatial-peak pulse-average intensity of $I_{sppa} \approx 32.5 W/cm^2$. Since the stimulation was continuous (duty cycle = 1), the spatial-peak

temporal-average intensity was $I_{spta} = I_{sppa} \approx 32.5W/cm^2$. We have added these values and the measurement conditions to the revised Methods and Discussion. We note that pressures and intensities at the cortical target may be different than water-tank measurements due to skull transmission, and constructive and destructive interference due to emergent standing waves, and we now clarify this point in the manuscript.

Added to methods:
'*Temperature recording experiments.* Temperature measurements were performed using a temperature probe positioned in the ultrasound coupling gel directly above the skull and immediately below the distal end of the ultrasound cone. A continuous acoustic signal was applied for 6 s and repeated six times, while temperature was recorded continuously. Ultrasound was delivered in continuous-wave (CW) mode at 500 kHz with a spatial-peak pressure amplitude of 1 MPa at the focus, as measured in water using a calibrated hydrophone (**Supplemental Note 1**). For a sinusoidal acoustic wave, the spatial-peak intensity is given by: $I = \frac{p_{pk}^2}{2\rho c}$, where p_{pk} is the peak pressure, ρ is the medium density, and c is the speed of sound. Using $\rho = 1000kg/m^3$ and $c = 1540m/s$, this corresponds to a spatial-peak pulse-average intensity of $I_{sppa} \approx 32.5W/cm^2$. Since stimulation was continuous (duty cycle = 1), the spatial-peak temporal-average intensity was, $I_{spta} = I_{sppa} \approx 32.5W/cm^2$, yielding a thermal index of approximately 1.4.'

Updated in the discussion:
'We note that the continuous-wave exposure used here exceeds the recommended thermal index (TI ≈ 1) for human diagnostic ultrasound [10,11] (TI ≈ 1.4; $\mathrm{I_{spta}} = \mathrm{I_{sppa}} \approx 32.5\mathrm{W/cm^2}$). Accordingly, translation of this approach to human applications will require waveform optimisation and careful consideration of skull-induced attenuation and aberration[12].'

Reviewer 2, Comment 5: Why does the amplitude of V_{AE} depend on the difference frequency? Is this physical or physiologic in origin? Can it be predicted based on theory?

Response: The dependence of V_{AE} amplitude on the difference frequency was observed consistently in both in vitro and in vivo experiments (**Fig. 2**) and was also reported in our prior acoustoelectric phantom study performed in a homogeneous saline medium, which isolates the interaction physics from physiology. These phantom experiments indicate that the frequency dependence is predominantly physical in origin, rather than arising from physiological processes.

From theory, the acoustoelectrically induced electric-field modulation can be described by

$$\nabla \cdot \vec{E}_{ae} = -k\overrightarrow{\nabla P} \cdot \vec{E}_o \qquad (1)$$

where $\overrightarrow{\nabla P}$ is the pressure gradient, $\vec{E}_o$ is the externally applied electric field, and $\vec{E}_{ae}$ is the resulting acoustoelectrically generated field modulation (derivation and phantom validation in prior work in Communications Physics[4]). This relation captures the proportional dependence of the acoustoelectric signal on acoustic and electric fields, but in its present form it does not predict the observed dependence of amplitude on the difference frequency (i.e., it does not specify a full frequency-domain transfer function for $V_{AE}\ (\Delta f)$.

Empirically, the Δf dependence could arise from one or more physical factors that shape the detected low-frequency component, including medium impedance, tissue/electrolyte dispersion, as well as spatial averaging effects that vary with the wavelength of the difference-frequency field. At present we cannot uniquely attribute the Δf-dependence to a single mechanism, and we therefore avoid over-interpreting it. We have revised the manuscript to state that the phenomenon

appears physical (supported by saline-phantom data) but that explaining and predicting V_{AE} (Δf) in vivo will require future systematic investigation.

Reviewer 2, Comment 6: What is the spatial dependency (location of US focus in brain) on evoked responses? Was the US focal spot moved one beam width away from motor cortex to determine whether the stimulation effect was reduced/eliminated?

Response: The acoustic disconnection control experiment demonstrates that ultrasound coupling into the brain is necessary for the observed effect. However, systematic spatial mapping of evoked responses by translating the ultrasound focus within the mouse brain was not feasible under the present conditions. At 500 kHz, the acoustic wavelength in brain tissue (~3 mm) which does not attenuate sufficiently within the dimensions of the mouse brain, and numerical simulations indicate the presence of standing waves and multiple pressure maxima within the cranial cavity (**Supplemental Note 4**). As a result, translating the nominal focal spot by approximately one beam width does not produce a well-isolated change in intracranial pressure distribution, limiting the interpretability of such a manipulation in the mouse model. Similar non-focal or weakly focal EMG responses at comparable acoustic frequencies in mice have been reported previously[13]. We therefore attribute the limited spatial specificity observed here to acoustic wavelength and skull-induced interference in the mouse, rather than to the underlying stimulation mechanism itself.

Reviewer 2, Comment 7: Please include something quantitative in the abstract. What are the take-home numbers, such as the threshold of AES or AES decreased the tFUS threshold by XX (e.g., US and E-field amplitudes, what is the spatial selectivity in mm)?

Response: We thank the reviewer for this suggestion. We have revised the abstract to include quantitative take-home values that define the operating regime of acoustoelectric neuromodulation, including the acoustic carrier frequency and pressure (500 kHz, 1 MPa), the measured in vivo acoustoelectric potential amplitude (≈9 mV), the corresponding order-of-magnitude focal electric field (~6 V/m; geometry dependent), and spatial confinement demonstrated in controlled phantom maps (~1.5 mm between extrema). We did not report a single activation threshold or “threshold reduction” because response probability and amplitude were strongly state-dependent during the lightening anaesthesia window, making monotonic threshold estimates unreliable in this model; instead, we report field magnitudes, frequencies, and spatial scales that directly characterise the stimulation regime.

The revised abstract text is provided below:

Abstract

‘Non-invasive brain stimulation offers therapeutic potential without surgery, yet existing electrical approaches lack spatial precision due to the long wavelengths of electric fields. Here we demonstrate acoustoelectric neuromodulation, a nonlinear interaction between applied acoustic and electric fields that generates spatially localized, low-frequency electric fields at the ultrasound focus. Using in vitro and in vivo rodent electrophysiology, we show motor-evoked responses that depend on both the amplitude and frequency of the acoustoelectric field, with controls excluding purely acoustic or electrical origins. In vivo measurements show acoustoelectric potentials of ≈9 mV, corresponding to estimated focal electric fields of ~6 V/m at 500 kHz and 1 MPa acoustic pressure, with ~1.5 mm extrema spacing demonstrated in phantom experiments. Importantly, we identify an acoustoelectric contribution to conventional ultrasound stimulation, arising from

interactions between ultrasound-induced electrical signals and propagating acoustic waves, establishing acoustoelectric neuromodulation as a distinct mechanism influencing ultrasound-based brain stimulation.'

Reviewer 2, Comment 8: What is the clinical potential of AES? What are the barriers?

Response: We thank the reviewer for raising this important point. The principal clinical potential of acoustoelectric stimulation (AES) lies in providing a mechanistically defined and externally controllable means of non-invasive neuromodulation, in which the amplitude and spatial localisation of the induced low-frequency electric field are jointly determined by independently applied acoustic and electric fields. This dual-field architecture offers two independent control axes for optimising stimulation efficacy while explicitly constraining safety-relevant parameters.

A key advantage of AES is that the induced field can be calibrated ex vivo, by measuring the spatial profile and magnitude of the acoustoelectrically generated electric field in tissue-mimicking phantoms, providing a level of predictability and mechanistic grounding that is difficult to achieve with existing non-invasive modalities. In this context, our results also suggest that acoustoelectric interactions may contribute to the effects observed in transcranial focused ultrasound (tFUS), raising the possibility that AES-informed waveform design could improve the specificity and safety of ultrasound-based neuromodulation.

The principal barriers to clinical translation include: (i) acoustic transmission through the human skull, including attenuation and phase aberration; (ii) thermal constraints, particularly for continuous-wave or high-duty-cycle operation; (iii) scaling spatial specificity from rodent to human brain geometries; and (iv) the need for integrated hardware capable of stable, reproducible delivery of coupled acoustic and electric fields under clinical safety standards and regulations. Addressing these challenges will require waveform optimisation, patient-specific acoustic modelling, and further validation in large animal models.

Potential long-term clinical applications include non-invasive alternatives to neurosurgical stimulation approaches for epilepsy[14], obsessive compulsive disorder[15], depression[16], Alzheimer's disease[17], and post-stroke neuroplasticity or neurogenesis[18]. However, we emphasise that translation will require careful engineering and safety optimisation rather than direct extrapolation from the present mouse experiments.

Reviewer 2, Comment 9: The peer-reviewed paper connected to reference #16 (Alvarez et al. 2020) is Alvarez et al. "In vivo acoustoelectric imaging for high-resolution visualization of cardiac electric spatiotemporal dynamics." Appl Opt. 2020 Dec 20;59(36):11292-11300. doi: 10.1364/AO.410172.

Response: We thank the reviewer for clarifying the scope of Alvarez et al. (2020). We agree that this work concerns cardiac acoustoelectric imaging rather than neural stimulation.

The citation was included to demonstrate the technical maturity and in vivo feasibility of ultrasound current-source-density imaging based on the acoustoelectric effect, particularly with respect to achieving millimetre-scale spatial localisation and high temporal resolution of bioelectric activity in living tissue. Although demonstrated in the cardiac context, this work provides a well-validated example of how acoustoelectric interactions can be exploited to localise electrical signals in vivo, which is directly relevant to the physical principles underlying acoustoelectric neuromodulation.

To avoid any possible confusion regarding the scope of this reference, we have clarified its role in the revised manuscript by explicitly framing it as an imaging example rather than a neuromodulation study. The following sentence has been updated in the Introduction:

'This principle underlies ultrasound current-source-density imaging[19,20], enabling millimetre-scale spatial mapping of current distributions in cardiac tissue using the acoustoelectric effect.'

This clarification ensures that the citation is interpreted as evidence of the spatial and temporal localisation capabilities of the acoustoelectric mechanism itself, rather than as prior work on neural stimulation.

Reviewer #2 (Remarks on code availability):

I verified the code is present and documented. I did not otherwise evaluate the code or files.

We thank the reviewer for verifying that the code is available and documented. We have ensured the Code availability statement clearly links to the repository (including documentation and dependencies) to support reproducibility.

Reviewer #3:

In this manuscript, Rintoul et al. demonstrate a non-invasive neuromodulation approach that combines electrical and mechanical (ultrasound) waves to achieve electrically focused stimulation. The authors propose that small, local ion conductivity changes driven by mechanical waves can induce voltage gradients in the presence of an applied electric field. Furthermore, this multimodal interaction can generate signals with frequencies equal to the difference between the principal waves, which may serve as a mechanism for neuromodulation. Overall, this concept is quite interesting from both physical and engineering perspectives. However, there are major concerns regarding the significance and accuracy of the results presented.

Comments

Reviewer 3, Comment 1: The primary motivation for establishing such a multimodal system is to achieve direct electrical stimulation with a spatial resolution comparable to focused ultrasound. In no part of the manuscript do the authors provide information regarding the in vivo spatiotemporal resolution of the stimulation. While the videos are informative, it is evident that the effects of stimulation are highly widespread, including bilateral motor twitches.

Response: We thank the reviewer for raising this important point. We agree that, in the present in vivo mouse experiments, the observed motor responses are spatially widespread, including bilateral movements, and do not demonstrate localized stimulation within the brain. We have clarified this limitation explicitly in the revised manuscript.

With respect to spatiotemporal resolution, the temporal structure of AES is determined by the imposed difference frequency Δf, and we demonstrate responses under Δf = 0 (DC onset-dominated) and Δf = 1 Hz conditions. Spatially, we quantify millimetre-scale confinement of the acoustoelectric field in controlled phantom experiments (≈1.5 mm between extrema at 500 kHz; **Fig. 2b–c**), but in vivo spatial focality in mouse at 500 kHz cannot be reliably inferred from EMG laterality or video behaviour.

The motivation for AES is twofold. First, AES provides a mechanistically defined means of generating low-frequency electric fields whose spatial distribution is governed by the ultrasound pressure field, offering the potential for improved spatial specificity relative to electrical stimulation alone when physical boundary conditions permit. Second, AES enables independent control of stimulation amplitude and safety-relevant parameters through separate tuning of the applied acoustic and electric fields. This latter capability is demonstrated in vivo, where response metrics can be modulated by varying either the applied electric field (**Figs. 3 and 6**) or the acoustic pressure (**Fig. 7**).

In the mouse brain, spatial focality is fundamentally limited by physical constraints. At 500 kHz, the acoustic wavelength in brain (~3 mm) is comparable to mouse head dimensions, and skull/air boundary reflections can generate standing waves and multiple intracranial pressure maxima. Finite-element simulations in a realistic mouse head model (**Supplementary Note 4**) demonstrate that these effects substantially diminish spatial confinement independent of stimulation mechanism; therefore, translating the nominal focus by one beam width would not produce a well-isolated change in intracranial pressure distribution.

To address spatial resolution under conditions where boundary effects are controlled, we rely on in vitro phantom mapping in a larger, acoustically damped volume, where the acoustoelectric field closely follows the ultrasound focus (**Fig. 2a–c; Supplementary Note 1e–g**). These experiments establish the intrinsic spatial selectivity of the acoustoelectric mechanism itself. We now clearly distinguish between (i) demonstrated in vivo physiological effects and controllability in the mouse model and (ii) the achievable focality of AES under conditions where wavelength and boundary constraints are favourable. We view higher ultrasound carrier frequencies or larger-animal models as necessary next steps to realize localized in vivo stimulation.

The limitations paragraph in the Discussion section has been updated to clarify this point:

‘Although acoustoelectric neuromodulation is motivated by the potential for spatially confined electric-field generation, the present in vivo mouse experiments do not demonstrate localized cortical stimulation, and the observed bilateral motor responses reflect physical constraints of the model rather than limitations of the underlying mechanism. At the ultrasound carrier frequency used here, the relationship between mouse head size and acoustic wavelength promotes non-focal standing waves (**Supplemental Note 4**). Using higher-frequency transducers or larger heads (e.g., humans) should reduce standing-wave artefacts and yield more focal acoustoelectric fields[21].’

And the results text in **Fig. 3** updated:
‘Although motor responses in **Fig. 3** and **Video Supplement 1** can appear unilateral (and in some cases bilateral), behavioral laterality cannot be taken as evidence of unilateral cortical target engagement under these conditions.’

Results text in **Fig. 4** updated at end of first paragraph to include:
‘As in **Fig. 3**, behavioral laterality/extent was not used to infer spatial focality in vivo at 500 kHz, because intracranial standing-wave patterns can produce multiple pressure maxima (**Supplementary Note 4**).’

Reviewer 3, Comment 2: Given the novelty of the experimental setup, clearer schematics and real experimental photographs would greatly improve the accessibility of the manuscript. The current schematic is confusing; the authors may consider developing a schematic centred on a coronal slice of the brain and skull.

Response: We have revised the schematic in **Supplemental Note S11** to be less confusing, with experimental photos added showing mouse head directly post-surgery, experimental set up and acoustoelectric stimulation experimental arrangement alongside a better picture of the Faraday cage and equipment used in acoustoelectric stimulation. The updated figure is included below for ease of review and has been incorporated into the revised Supplementary Information.

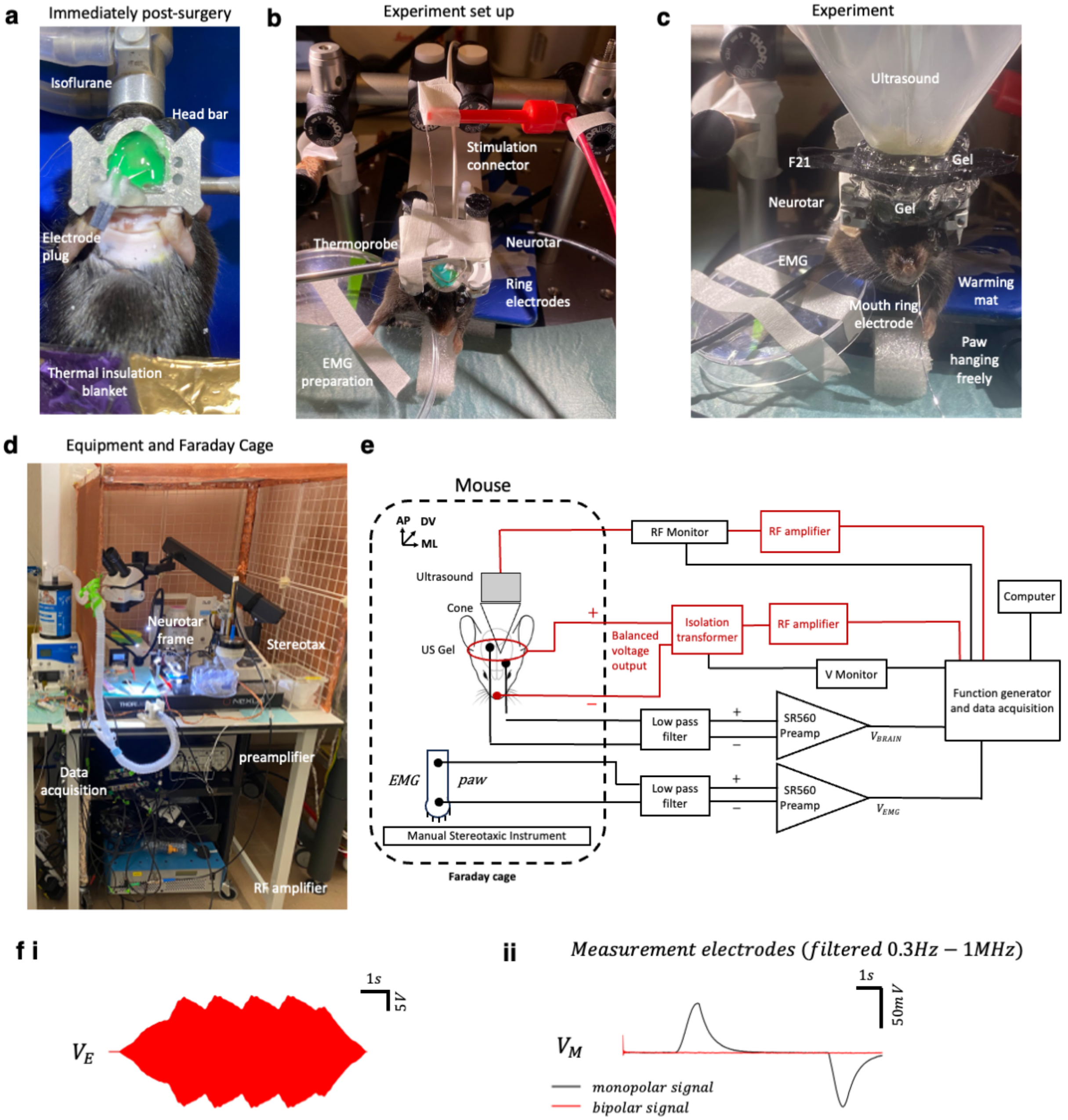

S11 | Acoustoelectric instrumentation. a, Electrodes implant and head bar installation immediately post-surgery **b,** Experiment preparation with stimulation ring electrode arrangement, and EMG preparation under Ketamine/Xylazine anaesthesia. **c,** Mouse during experiment with EMG measurement, ultrasound cone, gel and 2mm F21 in place. **d,** Photo of faraday cage showing Neurotar frame for mouse and hardware equipment below*.* ***e,*** Acoustoelectric neuromodulation instrumentation *block diagram* for acoustoelectric neuromodulation. Red lines show stimulation equipment and black lines show measurement system. ***f,*** i) Stimulation signal monitored during 70V peak-to-peak 500kHz voltage signal applied via the Platinum

Iridium electrodes used for stimulation in the mouse, some reverberation is present due to electric reflections. ii) Differential measurement at electrodes in 0.9% physiological saline phantom, sampling rate 5Mhz, with differential preamplifier front end filter 0.3Hz-1MHz, gain=1, 12 seconds duration showing monopolar arrangement DC offset at onset and offset (black line) and bipolar signal (red line) where both have been 40Hz software low-pass filtered to reveal the electrochemical DC drift induced between the stimulation electrodes. Note that the preamplifier front end filter was implemented (0.3Hz high pass) to keep the recorded signal within its saturation limits, meaning the recorded filtered signal shows the onset of the DC but not the actual offset.

Reviewer 3, Comment 3: In Figure 1b, the authors apply approximately 20 V (according to the scale bar) at f = 500 kHz, which results in no measurable changes in either LFP or EMG (E only). In contrast, in Figure 4a, a smaller voltage amplitude (6 V) applied at f = 500.001 kHz results in a measurable EMG response. Furthermore, similar stimulations (500Khz US) have very different EMG profile. Authors should carefully address these inconsistencies.

Response: We thank the reviewer for highlighting this apparent inconsistency. This point overlaps with **Reviewer 1, Comment 5**, and we refer the reviewer to that response for the full explanation and the corresponding manuscript edits.

The key point is that the response in **Fig. 4 a** is not driven by the high-frequency electric field alone (500.001 kHz), but by the low-frequency difference-frequency component (Δf = 1 Hz) generated only when the electric and acoustic fields are applied simultaneously. Accordingly, in the “E only” conditions (whether at 500 kHz or 500.001 kHz), no Δf component is generated in the absence of ultrasound, and the “E only” controls do not show a consistent 1 Hz spectral component in V_{BRAIN} or V_{EMG}} at the group level.

With respect to the apparent differences in EMG waveform shape between figures under otherwise similar ultrasound carrier frequency (500 kHz), **Figs. 3 and 4** were designed to test different mechanistic questions and therefore used different stimulation regimes (including different acoustic pressures). In addition, EMG responsiveness varies substantially with physiological state/anaesthesia depth over time. For this reason, the primary inference in both figures is based on within-triplet comparisons (counterbalanced, interleaved triplets with matched parameters within each experiment), rather than comparisons of single traces across figures. To avoid over-interpretation of trace-to-trace variability, we have revised the manuscript to describe the displayed single-trial traces as illustrative examples (rather than “representative”) and to emphasize that statistical conclusions are drawn from the within-triplet, group-level analyses (**Supplementary Note 3**; consistent with prior mouse tFUS reports[9]).

Text added/clarified in **Fig. 4** Results: “Notably, the high-frequency electric field alone (500.001 kHz) does not generate a difference frequency; the measurable EMG modulation arises from the low-frequency difference-frequency field (Δf = 1 Hz) produced only when the acoustic and electric fields are applied simultaneously.”

Reviewer 3, Comment 4: Despite the inclusion of multiple control experiments, the current results do not conclusively demonstrate effective local stimulation. Given the novelty of this approach, additional neural spiking analyses—such as post-stimulus time histograms of unit firing and coherence between firing rates and stimulation frequencies—are necessary to support the claims.

We thank the reviewer for this thoughtful comment and agree that direct spiking analyses (e.g., PSTHs, spike–stimulus coherence) would provide stronger evidence of neural encoding and would be an important direction for future work. In the present study, however, our experimental

paradigm was designed to test the physics and controllability of acoustoelectric field generation during an acute stimulation window under ketamine/xylazine, rather than to obtain stable unit recordings. Implementing spiking analyses would require a substantially different preparation (stable single-/multi-unit recordings with spike sorting, longer recording stability, and stimulation schedules compatible with unit isolation), which was not feasible under the present setup and time window.

Importantly, our central claims are therefore limited to: (i) demonstrating focal acoustoelectric field generation and frequency dependence in controlled phantoms (**Fig. 2; Supplementary Note 1**), and (ii) showing in vivo physiological effects and artefact controls consistent with an acoustoelectric contribution (**Figs. 3–7**). With respect to spatial localisation, we explicitly distinguish between what is achievable under controlled boundary conditions (phantoms) and what is physically achievable in the mouse head at 500 kHz, where wavelength-scale standing waves limit spatial isolation (**Supplementary Note 4**). We have revised the manuscript to clarify this scope and to avoid implying single-unit entrainment or localized in vivo cortical stimulation under these conditions.

We have added the following sentence to the Limitations paragraph in the Discussion: "Direct neural spiking analyses (e.g., post-stimulus time histograms or spike–stimulus coherence) would require stable single-unit or multi-unit recordings under conditions not compatible with the present acute stimulation paradigm and are therefore left to future studies.'

Reviewer 3, Comment 5: What is the magnitude of the ion concentration changes induced by the mechanical waves? Based on the data presented for acoustoelectric stimulation, the resulting electric field appears to be on the order of hundreds of microvolts, which is significantly lower than what is typically required for neural entrainment.

Response: We thank the reviewer for this important question. The mechanical waves used here do not induce large or sustained bulk ion concentration changes. Rather, the acoustoelectric effect arises from small, transient modulations of ionic mobility and effective electrical conductivity that occur during the acoustic compression–rarefaction cycle, rather than from net ion transport, accumulation, or DC concentration gradients. These perturbations are physical in origin and have been characterised previously in homogeneous conductive media using acoustoelectric phantom experiments[4].

From theory, the induced acoustoelectric field is described by the governing relation

$$\nabla \cdot \vec{E}_{ae} = -k\overrightarrow{\nabla P} \cdot \vec{E}_o \qquad (1)$$

where $\overrightarrow{\nabla P}$ is the pressure gradient, $\vec{E}_o$ is the externally applied electric field, and $\vec{E}_{ae}$ is the acoustoelectrically induced electric-field modulation. The proportionality constant k captures pressure-dependent changes in ionic mobility and compressibility; a full derivation is provided in the Supplementary Material of our prior work in Communications Physics[4]. Consistent with earlier experimental and theoretical studies[22], ionic mobility effects dominate the acoustoelectric response in conductive biological media. Absolute ion concentration changes are therefore expected to be very small and oscillatory and cannot be meaningfully quantified in vivo given tissue heterogeneity and anisotropy.

Using the in vivo measurements in **Fig. 3** to estimate neuromodulation feasibility, we observe a difference-frequency acoustoelectric potential of approximately ≈9mV peak onset; **Fig. 3 b**). From spatial acoustoelectric maps (**Fig. 2 b,c**), the separation between extrema corresponds to

approximately half the acoustic wavelength (≈1.5 mm at 500 kHz), yielding an estimated local electric-field magnitude of ~6 V/m at the ultrasound focus. Thus, while the measured voltage differences are on the order of a millivolt, the relevant local field strength experienced by neural tissue is several volts per meter, not hundreds of microvolts per meter.

Importantly, AES is not intended to replicate the broadly distributed fields used in tACS/tDCS or the large induction fields associated with TMS. Instead, AES generates a spatially confined, low-frequency electric field via acoustoelectric mixing, whose magnitude is determined by the local conductivity and acoustic pressure gradient. This localized gradient field interacts with neural elements over millimetre length scales, distinguishing AES from conventional electrical stimulation modalities which work over larger length scales and provides a mechanistically grounded route to neuromodulation without requiring large bulk ion concentration changes.

Overall, this is a very intriguing finding; however, the current data do not adequately support the stated claims, and multiple additional experiments are required to substantiate the conclusions.

Response: We thank the reviewer for this assessment. In response, we have revised the manuscript to more precisely scope our claims to: (i) establishing the physical basis and spatial selectivity of acoustoelectric mixing in controlled phantoms (**Fig. 2 and Supplementary Note 1**), (ii) demonstrating in vivo physiological effects under an acute stimulation paradigm using counterbalanced, interleaved controls (**Figs. 3–4**), and (iii) quantifying key artefact controls and parameter controllability, including transducer-induced electrical coupling and its attenuation (**Figs. 5–7**). We now explicitly state the limitations of in vivo spatial confinement at 500 kHz in the mouse model due to wavelength-scale and boundary effects (**Supplementary Note 4**), and we clarify that single-unit spiking analyses, systematic activation curves, and spatial response mapping would require substantially different experimental paradigms and are therefore left to future work. We believe these revisions align the conclusions with what is directly supported by the present dataset while clearly identifying the most important next experimental steps.

REFERENCES:

1. King, R. L., Brown, J. R., Newsome, W. T. & Pauly, K. B. Effective parameters for ultrasound-induced in vivo neurostimulation. *Ultrasound Med. Biol.* **39**, 312–331 (2013).
2. Rattay, F. Analysis of Models for External Stimulation of Axons. *IEEE Trans. Biomed. Eng.* **BME-33**, 974–977 (1986).
3. Schmid, S., Wilson, D. A. & Rankin, C. H. Habituation mechanisms and their importance for cognitive function. *Front. Integr. Neurosci.* **8**, 97 (2015).
4. Rintoul, J. L., Neufeld, E., Butler, C., Cleveland, R. O. & Grossman, N. Remote focused encoding and decoding of electric fields through acoustoelectric heterodyning. *Communications Physics 2023 6:1* **6**, 1–11 (2023).
5. Jossinet, J., Lavandier, B. & Cathignol, D. The phenomenology of acousto-electric interaction signals in aqueous solutions of electrolytes. *Ultrasonics* **36**, 607–613 (1998).
6. Yuan, Y., Wang, X., Yan, J. & Li, X. The effect of anesthetic dose on the motor response induced by low-intensity pulsed ultrasound stimulation Shanbao Tong, Junfeng Sun. *BMC Neurosci.* **19**, 1–6 (2018).
7. Opitz, A. *et al.* Physiological observations validate finite element models for estimating subject-specific electric field distributions induced by transcranial magnetic stimulation of the human motor cortex. *Neuroimage* **81**, 253–264 (2013).
8. Shahid, S., Wen, P. & Ahfock, T. Assessment of electric field distribution in anisotropic cortical and subcortical regions under the influence of tDCS. *Bioelectromagnetics* **35**, 41–57 (2014).

9. Yuan, Y., Wang, X., Yan, J. & Li, X. The effect of anesthetic dose on the motor response induced by low-intensity pulsed ultrasound stimulation. *BMC Neurosci.* **19**, 78 (2018).
10. Duck, F. A. The Meaning of Thermal Index (TI) and Mechanical Index (MI) Values. *Ultrasound* **5**, 36–40 (1997).
11. Retz, K. *et al.* Measured acoustic intensities for clinical diagnostic ultrasound transducers and correlation with thermal index. *Ultrasound in Obstetrics & Gynecology* **50**, 236–241 (2017).
12. Tsai, P. C., Gougheri, H. S. & Kiani, M. Skull Impact on the Ultrasound Beam Profile of Transcranial Focused Ultrasound Stimulation. *Proceedings of the Annual International Conference of the IEEE Engineering in Medicine and Biology Society, EMBS* 5188–5191 (2019) doi:10.1109/EMBC.2019.8857269.
13. Mueller, J. K., Ai, L., Bansal, P. & Legon, W. Numerical evaluation of the skull for human neuromodulation with transcranial focused ultrasound. *J. Neural Eng.* **14**, 066012 (2017).
14. Jobst, B. C. & Cascino, G. D. Resective Epilepsy Surgery for Drug-Resistant Focal Epilepsy: A Review. *JAMA* **313**, 285–293 (2015).
15. Luyten, L., Hendrickx, S., Raymaekers, S., Gabriëls, L. & Nuttin, B. Electrical stimulation in the bed nucleus of the stria terminalis alleviates severe obsessive-compulsive disorder. *Molecular Psychiatry 2016 21:9* **21**, 1272–1280 (2015).
16. Holtzheimer, P. E. & Nemeroff, C. B. Advances in the treatment of depression. *NeuroRx* **3**, 42–56 (2006).
17. Chang, C. H., Lane, H. Y. & Lin, C. H. Brain stimulation in Alzheimer's disease. *Front. Psychiatry* **9**, 329705 (2018).
18. Sharififar, S., Shuster, J. J. & Bishop, M. D. Adding electrical stimulation during standard rehabilitation after stroke to improve motor function. A systematic review and meta-analysis. *Ann. Phys. Rehabil. Med.* **61**, 339–344 (2018).
19. Olafsson, R. *et al.* Cardiac activation mapping using ultrasound current source density imaging (UCSDI). *IEEE Trans. Ultrason. Ferroelectr. Freq. Control* **56**, 565–574 (2009).
20. Alvarez, A., Preston, C., Trujillo, T. & Witte, R. S. Acoustoelectric imaging for beat-to-beat cardiac activation wave mapping in an in vivo swine model. *IEEE International Ultrasonics Symposium, IUS* **2020-September**, (2020).
21. Martin, E. *et al.* Ultrasound system for precise neuromodulation of human deep brain circuits. *Nature Communications 2025 16:1* **16**, 1–14 (2025).
22. Lavandier, B., Jossinet, J. & Cathignol, D. Quantitative assessment of ultrasound-induced resistance change in saline solution. *Med. Biol. Eng. Comput.* **38**, 150–155 (2000).
